# Genetic compensation prevents myopathy and heart failure in an *in vivo* model of Bag3 deficiency

**DOI:** 10.1371/journal.pgen.1009088

**Published:** 2020-11-02

**Authors:** Federica Diofano, Karolina Weinmann, Isabelle Schneider, Kevin D. Thiessen, Wolfgang Rottbauer, Steffen Just

**Affiliations:** 1 Molecular Cardiology, Department of Internal Medicine II, University of Ulm, Ulm, Germany; 2 Department of Internal Medicine II, University of Ulm, Ulm, Germany; Max-Planck-Institut fur Herz- und Lungenforschung W G Kerckhoff-Institute, GERMANY

## Abstract

Mutations in the molecular co-chaperone Bcl2-associated athanogene 3 (BAG3) are found to cause dilated cardiomyopathy (DCM), resulting in systolic dysfunction and heart failure, as well as myofibrillar myopathy (MFM), which is characterized by protein aggregation and myofibrillar disintegration in skeletal muscle cells. Here, we generated a CRISPR/Cas9-induced Bag3 knockout zebrafish line and found the complete preservation of heart and skeletal muscle structure and function during embryonic development, in contrast to morpholino-mediated knockdown of Bag3. Intriguingly, genetic compensation, a process of transcriptional adaptation which acts independent of protein feedback loops, was found to prevent heart and skeletal muscle damage in our Bag3 knockout model. Proteomic profiling and quantitative real-time PCR analyses identified Bag2, another member of the Bag protein family, significantly upregulated on a transcript and protein level in *bag3*^*-/-*^ mutants. This implied that the decay of *bag3* mutant mRNA in homozygous *bag3*^*-/-*^ embryos caused the transcriptional upregulation of *bag2* expression. We further demonstrated that morpholino-mediated knockdown of Bag2 in *bag3*^*-/-*^ embryos evoked severe functional and structural heart and skeletal muscle defects, which are similar to Bag3 morphants. However, Bag2 knockdown in *bag3*^*+/+*^ or *bag3*^*+/-*^ embryos did not result in (cardio-)myopathy. Finally, we found that inhibition of the nonsense-mediated mRNA decay (NMD) machinery by knockdown of *upf1*, an essential NMD factor, caused severe heart and skeletal muscle defects in *bag3*^*-/-*^ mutants due to the blockade of transcriptional adaptation of *bag2* expression. Our findings provide evidence that genetic compensation might vitally influence the penetrance of disease-causing *bag3* mutations *in vivo*.

## Author summary

One form of genetic compensation is described as transcriptional adaptation of gene expression triggered by deleterious gene mutations. Although the precise molecular mechanism that induces genetic compensation needs to be defined, it represents a powerful biological phenomenon that warrants genetic robustness. We find that antisense-mediated knockdown of Bag3 in zebrafish embryos causes heart failure and myopathy. By contrast, CRISPR/Cas9-induced depletion of Bag3 does not result in the abrogation of heart and skeletal muscle function in zebrafish embryos. We find here that transcriptional activation of the Bag family member *bag2* is capable of restoring heart and skeletal muscle function in *bag3* mutant embryos, whereas this compensatory mechanism is not present in the *bag3* morphants. Furthermore, we show that nonsense-mediated decay of *bag3* mRNA is the molecular trigger for the compensatory upregulation of *bag2*. Our study provides evidence that genetic compensation via transcriptional adaptation is a vital modulator of disease peculiarity and penetrance in *bag3* mutant zebrafish and that this biological phenomenon might also be active in certain human BAG3 mutation carriers.

## Introduction

Compensatory transcriptional adaptation of gene expression in response to malignant and harmful gene mutations is a powerful mechanism to warrant genetic robustness. Interestingly, this genetic compensation was recently demonstrated to be triggered by a decay of the mutated mRNA which results in the transcriptional upregulation of one or more related and compensation-competent genes [1–3]. Recently, Rossi and coworkers found that CRISPR/Cas9-induced mutation of *egfl7*, an endothelial ECM gene, did not result in obvious defects in zebrafish. Whereas morpholino-modified antisense oligonucleotide (morpholino, MO)-mediated *egfl7* depletion led to severe vascular malformations [3]. Interestingly, Emilins, also important ECM genes, were found to be transcriptionally upregulated in *egfl7* mutant zebrafish, but not in morphant embryos, suggesting their compensatory potential to buffer the organism to *egfl7* loss. Additionally, Sztal et al. recently found genetic compensation in a zebrafish model of Actin deficiency [4]. Genetic *actc1b* mutant zebrafish embryos only show very mild muscle defects due to transcriptional upregulation and functional compensation by an α-Actin paralogue. Interestingly, morpholino-mediated *actc1b* depletion did not result in compensatory upregulation of other Actin family members thereby leading to severe myopathy *in vivo* [4].

B-cell lymphoma 2 (Bcl2)-associated athanogene 3 (BAG3) is a member of the BAG protein family which acts as a molecular co-chaperone by physically interacting with chaperone molecules such as 14-3-3 proteins, Hsp70, and small heat shock proteins (HSPBs) [5]. One of the main biological functions of BAG3 is the regulation of autophagy and the degradation of misfolded proteins to warrant orchestrated protein homeostasis [5, 6]. Mammalian BAG3 is strongly expressed in the heart and skeletal muscle, where it mainly localizes to sarcomeric Z-disks, but is also expressed in the brain and peripheral nervous system [7]. Mutations in the Bag3 gene are described to cause a diverse spectrum of disease phenotypes including striated muscle diseases such as dilated cardiomyopathy (DCM) or myofibrillar myopathy (MFM). Furthermore, decreased BAG3 protein levels were found in failing human hearts [8].

In this study, we uncovered and explored an example of functional genetic compensation in a CRISPR/Cas9-induced zebrafish model of Bag3-deficiency. We and others found that targeted morpholino-mediated knockdown of Bag3 in zebrafish embryos resulted in severe structural and functional heart and skeletal muscle defects [9–11]. Interestingly and in contrast to these studies, we found here that the CRISPR/Cas9-induced deletion of 19 nucleotides in exon 2 of the *bag3* gene leading to a frame shift and thereby introducing a premature stop codon did not evoke heart and skeletal muscle defects in homozygous mutant embryos. We determined that the degradation of mutated *bag3* mRNA triggered the compensatory transcriptional upregulation of the BAG protein family member *bag2*, which was able to functionally compensate for the loss of Bag3.

Our findings present another example of genetic compensation *in vivo*, underlining the importance of this exciting biological mechanism in buffering the malignancy of specific gene mutations and, thereby, mediating genetic robustness.

## Results

### Targeted knockout of zebrafish *bag3* by CRISPR/Cas9 does not provoke heart and skeletal muscle dysfunction

In humans, mutations in BAG3 (Bcl2-associated athanogene 3) were found to cause dilated cardiomyopathy (DCM) and myofibrillar myopathies (MFM) [9, 12]; however, the molecular BAG3-associated underpinnings are poorly understood. To study the *in vivo* roles of BAG3 in more detail, we generated a Bag3-deficient zebrafish line by using the CRISPR/Cas9 genome-editing technology.

Although teleosts went through whole genome duplication during evolution [13, 14], only one single *bag3* ortholog of the human BAG3 gene can be found on zebrafish chromosome 13. Zebrafish Bag3 shows 41% overall amino acid homology to human BAG3, with up to 77% sequence identity in distinct functional domains such as the WW domain (S1 Fig). As in humans, zebrafish *bag3* transcripts are enriched in the heart and skeletal muscle during embryogenesis and are more predominantly expressed in the adult cardiac muscle [10, 15].

**Fig 1:**
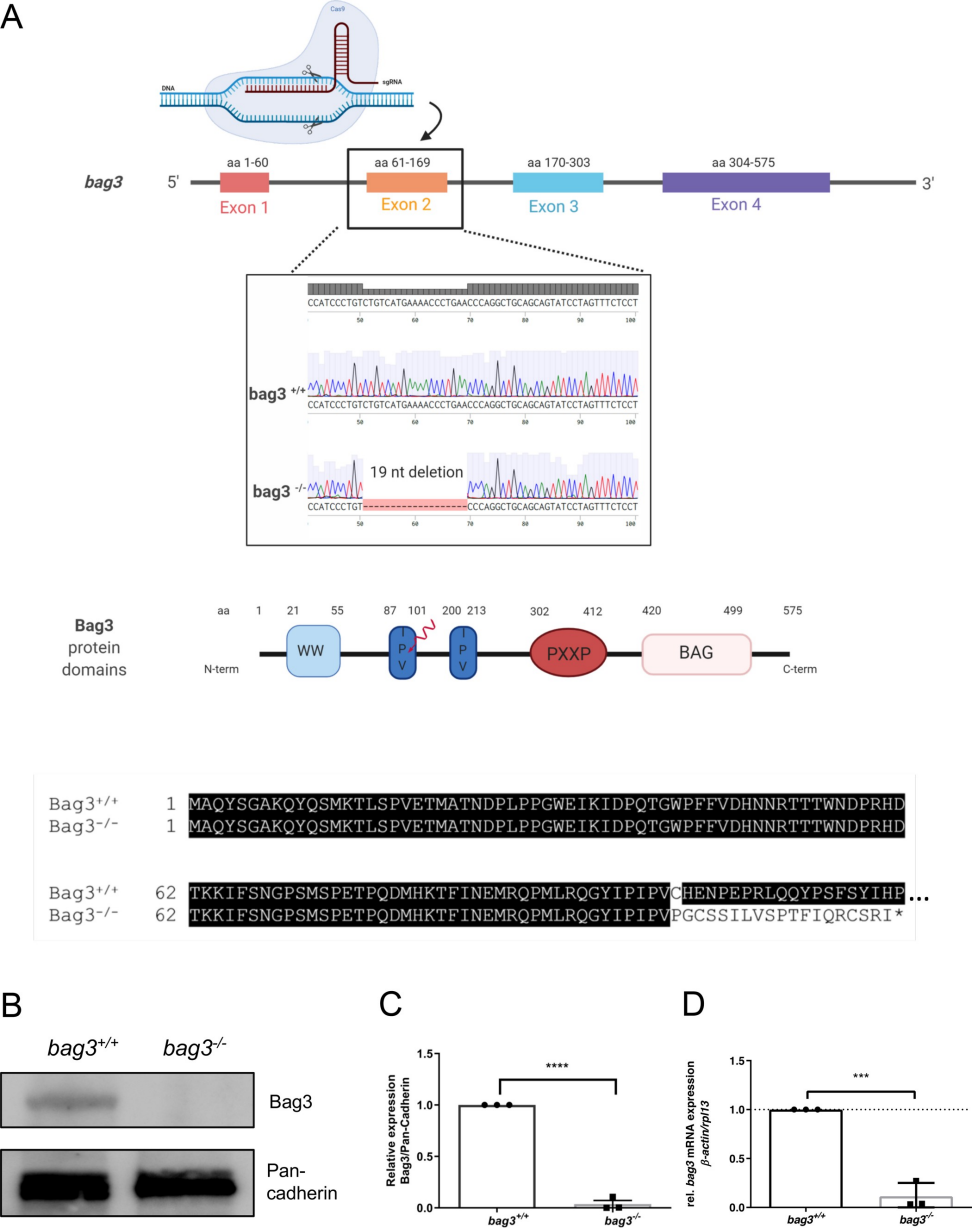
Generation of zebrafish *bag3* knockout by CRISPR/Cas9 gene editing. **(A)** Structure of the zebrafish *bag3* gene and protein. Exon 2 is the target for the CRISPR/Cas9 gene editing in zebrafish *bag3*. The CRISPR/Cas9-induced mutation (19 bp deletion) in *bag3* is shown in *bag3* mutant DNA sequencing chromatogram. The 19 nucleotides deletion in *bag3*^*-/-*^ leads to a frame shift, the introduction of a premature stop codon and thereby the premature termination of Bag3 translation, as demonstrated by the alignment of the Bag3^-/-^ and Bag3^+/+^ aminoacid sequences (only partial aminoacid sequence shown) **(B-C)** Immunoblot analysis of 72 hpf *bag3*^*+/+*^ embryo protein lysates compared to lysates obtained from *bag3*^*-/-*^ clutchmates with antibody against zebrafish Bag3. The figure shows one representative immunoblot from three independent experiments (N = 3, mean ± SD, P<0.0001 determined using two-tailed t-test). **(D)** Quantitative real-time PCR of *bag3*^*+/+*^ and *bag3*^*-/-*^ embryos at 72 hpf shows significant downregulation of *bag3* mRNA levels in *bag3*^*-/-*^ embryos (N = 3, mean ± SD, P = 0.0004 determined using two-tailed t-test).

To generate a loss-of-function zebrafish model by introducing CRISPR/Cas9-induced mutations in the *bag3* gene, we first designed a CRISPR-RNA (crRNA) targeting exon 2 of the *bag3* genomic sequence. By co-injecting synthetic tracrRNA, *bag3*-specific crRNA, and Cas9 protein, a frameshift mutation in the *bag3* gene was generated, leading to an alternative stop codon and premature termination of protein translation. Specifically, a mutant allele with a 19-nucleotide deletion was identified among F1 fish and the mutant line was propagated (Fig 1A). The putative mutation leads to the loss of the crucial Bag3 protein domains IPV and PXXP as well as the BAG domain (Fig 1A). To confirm Bag3 deficiency, we performed immunoblot assays and found the complete loss of wild-type Bag3 proteins in homozygous mutant *bag3*^*-/-*^ embryos (N = 3, P<0.0001) (Fig 1B and 1C). We also performed quantitative RT-PCR analysis using homozygous mutant *bag3*^*-/-*^ embryos and found *bag3* mRNA levels significantly downregulated (N = 3, P<0.0001) (Fig 1D). This suggested that nonsense-mediated mRNA decay (NMD) was triggered by the CRISPR/Cas9-induced gene mutation.

**Fig 2:**
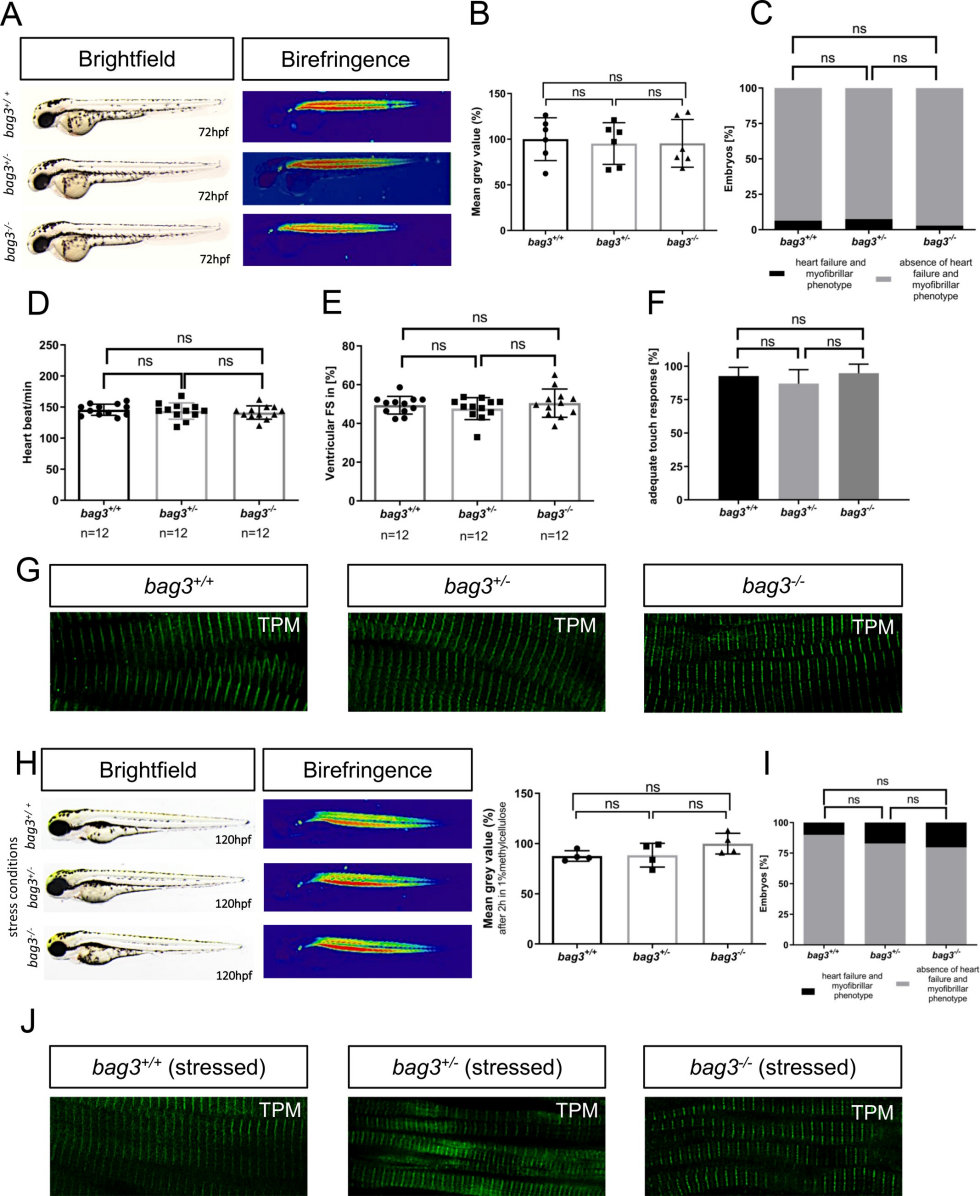
Genetic loss of *bag3* does not interfere with heart and skeletal muscle function. **(A)** Lateral view of brightfield and birefringence images for *bag3*^*+/+*^, *bag3*^*+/-*^ and *bag3*^*-/-*^ embryos at 72 hpf exhibiting homogenous birefringence signals. **(B)** Densitometric analysis of birefringence does not report muscle disorganization in *bag3*^*-/-*^ embryos. Representative samples are shown (n = 6, One-way ANOVA followed by tukey's multiple comparison analysis, P = 0.9688). **(C)** Embryos at 72 hpf reveal no myopathic phenotype (N = 3, n = 100, two-tailed value for Fisher´s exact test. *bag3*^*+/+*^ and *bag3*^*+/-*^ P = 0.8096; *bag3*^*+/+*^ and *bag3*^*-/-*^ P = 0.4949; *bag3*^*+/-*^ and *bag3*^*-/-*^ P = 0.1925). **(D)** Heart rate quantification at 72 hpf reveals no impairments under physiological conditions (N = 3, n = 12, mean ± S.D. One-way ANOVA followed by tukey's multiple comparison analysis, P = 0.6026). **(E)** Quantification of ventricular FS at 72 hpf does not reveal contractile dysfunction (N = 3, n = 12, mean ± S.D. One-way ANOVA followed by tukey's multiple comparison analysis, P = 0.5061). **(F)** Quantification of the touch evoked assay. *bag3*^*-/-*^ embryos reveal no significant difference in responsiveness upon mechanical stimulus (N = 3, n = 90, mean ± S.D. One-way ANOVA followed by tukey's multiple comparison analysis, P = 0.7851). **(G)** Immunostaining of *bag3*^*+/+*^, *bag3*^*+/-*^ and *bag3*^*-/-*^ embryos at 72 hpf, with sarcomeric Tropomyosin, showing no muscle fiber disruptions. **(H)** Brightfield and birefringence images of mutant embryos at 120 hpf under stressed conditions reveal no myopathic phenotype, which was confirmed by densitometric analysis of birefringence signals. Representative samples are shown (n = 4, One-way ANOVA followed by tukey's multiple comparison analysis, P = 0.1824). **(I)** Embryos, under stressed conditions, do not develop myopathic phenotype (N = 4, n = 50, two-tailed value for Fisher´s exact test. *bag3*^*+/+*^ and *bag3*^*+/-*^ P = 0.6050*; bag3*^*+/+*^ and *bag3*^*-/-*^ P = 0.8320; *bag3*^*+/-*^ and *bag3*^*-/-*^ P = 0.8582). **(J)** Immunostaining of *bag3*^*+/+*^, *bag3*^*+/-*^ and *bag3*^*-/-*^ embryos at 120 hpf under stressed conditions (2 hours in 1% methylcellulose), with sarcomeric Tropomyosin, showing no muscle fiber disruptions.

In-crosses of heterozygous carriers yielded offspring demonstrating the regular mendelian genotypic ratio of 25% homozygous wild-type (*bag3*^*+/+*^), 50% heterozygous (*bag3*^*+/-*^) and 25% homozygous mutant (*bag3*^*-/-*^) embryos (N = 3, n = 100) (Fig 2A). Interestingly, none of the zebrafish embryos displayed morphological malformations of the heart or skeletal muscle [16] (Fig 2A–2C). Since the high organization of striated muscle tissue is able to polarize light, the myofibrillar disorganization within muscle tissue can therefore be easily visualized by a reduction in the birefringence signal intensity. In *bag3*^*-/-*^ embryos, birefringence signal intensity from the skeletal muscle was similar to the intensity in *bag3*^*+/-*^ and *bag3*^*+/+*^, demonstrating preserved muscle structure in Bag3-deficient zebrafish embryos (Fig 2B). Additionally, *bag3*^*-/-*^ embryos did not develop functional heart or skeletal muscle defects under normal conditions as demonstrated by the assessment of the heart rate (N = 3, n = 12, P = 0.6026. HR *bag3*^*+/+*^: 146±9.03 heart beat/min; HR *bag3*^*+/-*^: 144±13.12 heart beat/min; HR *bag3*^*-/-*^: 141±10.71 heart beat/min, at 72 hpf), contractile force by fractional shortening (FS) measurements (N = 3, n = 12, P = 0.5061. FS *bag3*^*+/*+^: 49.41±4.57%; FS *bag3*^*+/-*^: 47.64±5.67%; FS *bag3*^*-/-*^: 50.47±7.291%, at 72 hpf), and touch evoked assays (N = 3, n = 90, P = 0.7851. Mot *bag3*^*+/+*^: 92.67±3.48%; Mot *bag3*^*+/-*^: 86.99±4.54%; Mot *bag3*^*-/-*^: 94.7±3.34%, at 72 hpf) (Fig 2D–2F; S1–S8 Movies). Muscle structure in *bag3*^*+/-*^ and *bag3*^*-/-*^ embryos was indistinguishable from that of *bag3*^*+/+*^ embryos as demonstrated by Tropomyosin immunostainings (Fig 2G). Additionally, transmission electron microscopy (TEM) analyses of heart and skeletal muscle ultrastructure revealed no alterations between wild-type and *bag3*^*-/-*^ sarcomeric organization (S3A Fig). We found highly organized myofilaments in *bag3*^*-/-*^ mutant embryos with thin and thick filaments in well-aligned bundles and discernible A-, I-, M-bands and Z-disks. Moreover, as shown by immunostainings and confocal imaging, atrial and ventricular cardiomyocytes of *bag3*^*-/-*^ mutant embryos express myosin heavy chains (MHC) in a proper cardiac chamber specific manner, suggesting regular heart chamber specification (S3B Fig).

Since Bag3 loss did not result in phenotypic alterations during development under normal physiological conditions, we aimed to mechanically overload the skeletal muscle in Bag3-deficient zebrafish embryos. Mutant *bag3*^*-/-*^ embryos were incubated in 1% methylcellulose, leading to increased viscosity of the media, which is known to significantly enhance workload on skeletal muscle [10]. Interestingly, increased muscle workload resulted in no significant elevation of myopathic phenotypes in *bag3*^*-/-*^ embryos compared to *bag3*^*+/-*^ and *bag3*^*+/+*^ clutchmates (N = 4, n = 50, *bag3*^*+/+*^ and *bag3*^*+/-*^ P = 0.6050; *bag3*^*+/+*^ and *bag3*^*-/-*^ P = 0.8320; *bag3*^*+/-*^ and *bag3*^*-/-*^ P = 0.8582). (Fig 2H). Similar to our observations on unstressed *bag3* mutant embryos, methylcellulose-incubated *bag3*^*+/+*^, *bag3*^*+/-*^, and *bag3*^*-/-*^ embryos did not display structural failure of myofibers following treatment (Fig 2J). This demonstrates that loss of Bag3 in our CRISPR/Cas9-induced zebrafish line does not cause cardiomyopathy and skeletal muscle dysfunction under physiological or stressed conditions. Additionally, we found that homozygous mutant *bag3*^*-/-*^ fish can be raised to adulthood and they are fertile. However, we found a significant increase in the mortality rate of *bag3*^*-/-*^ zebrafish starting from 9 month of age as depicted in a Kaplan-Meier survival curve (S2 Fig), which is consistent with the recently published findings by Ding et al[17].

### Morpholino-mediated ablation of Bag3 specifically results in (cardio)-myopathy

**Fig 3:**
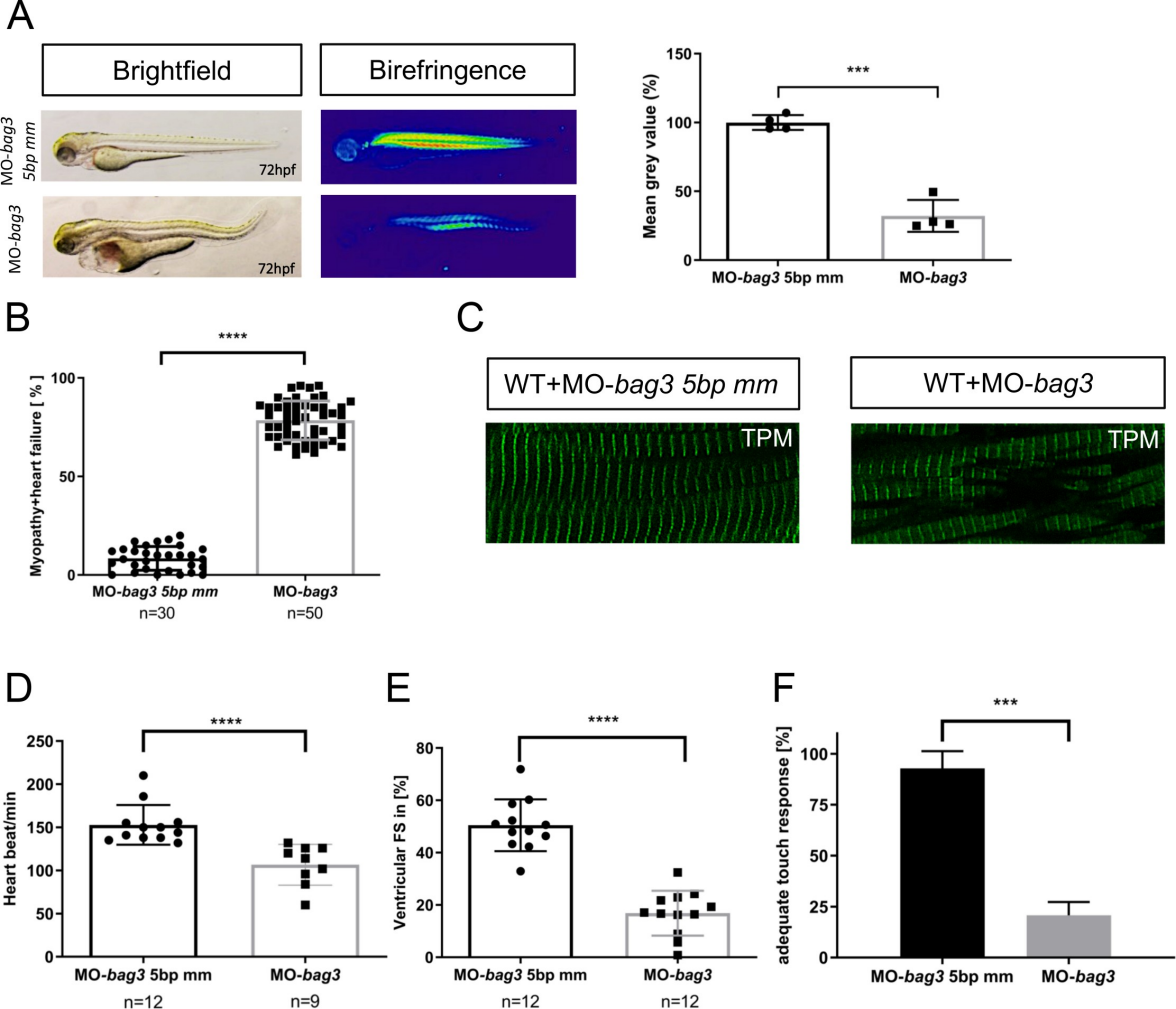
**Targeted knock-down of Bag3 leads to (cardio-)myopathy in zebrafish (A)** Brightfield and birefringence images of MO-*bag3* and MO-*bag3* 5bp mm injected embryos developed (cardio-)myopathy phenotype at 72 hpf. The densitometric analysis of birefringence supports the presence of myopathy phenotype only in *bag3* morphants. Representative samples are shown (n = 4, P = 0.0003 determined using two tailed t-test). **(B)** *bag3* splice MO injected embryos develop (cardio-)myopathy phenotype (78.42±9.88%) whereas control-injected embryos developed no pathological phenotype (78.42±9.89%) (N = 3, n = 30/50 mean± SD P<0.0001 determined using two-tailed t-tests). **(C)** Tropomyosin immunostaining of MO-*bag3* and MO-*bag3* 5bp mm embryos at 72 hpf shows that embryos injected with Bag3 splice MO develop muscle fiber disruptions. **(D)** Heart rate quantification of *bag3* morphants reveals impairments at 72 hpf (N = 3, n = 9/12, P = 0.6026. HR 5bp-mismatch-MO injected embryos: 153±22.98 heart beat/min; HR *bag3* morphants: 106±23.58 heart beat/min; mean ± S.D. P<0.0001 determined using two-tailed t-tests). **(E)** FS of *bag3* morphant ventricles at 72 hpf is significantly reduced (16,88±8.56%), compared to MO-*bag3* 5bp mm injected embryos (FS: 50.48±9.90%) (N = 3, n = 12; Mean± SD P<0.0001 determined using two-tailed t-tests). **(F)** Touch evoked assay reveals significant reduction in responsiveness upon mechanical stimulus for *bag3* morphants (20.78±4.46%) and not for 5bp-mismatch-MO injected embryos (92.78±4.40%) (N = 3, n = 40, mean ± S.D. P = 0.0005, determined using two-tailed t-tests).

Recently, different morpholino-mediated knockdown studies in zebrafish showed that loss of Bag3 led to severe heart and skeletal muscle defects and (cardio-)myopathy [9, 10, 18]. Similarly, we found severe cardiac and skeletal muscle defects (Fig 3A and 3B) associated with myofibrillar disorganization (Fig 3A–3C), diminished cardiac contractile function (Fig 3D and 3E; S9 Movie), and significantly reduced motility (Fig 3F) in zebrafish embryos injected with a morpholino targeting the splice donor site of exon 2 in *bag3* [9]. By contrast, injection of a specific 5bp mismatch control morpholino (MO-*bag3* 5bp mm) unable to target *bag3* did not evoke any cardiac and skeletal muscle defects (Fig 3A–3F).

**Fig 4:**
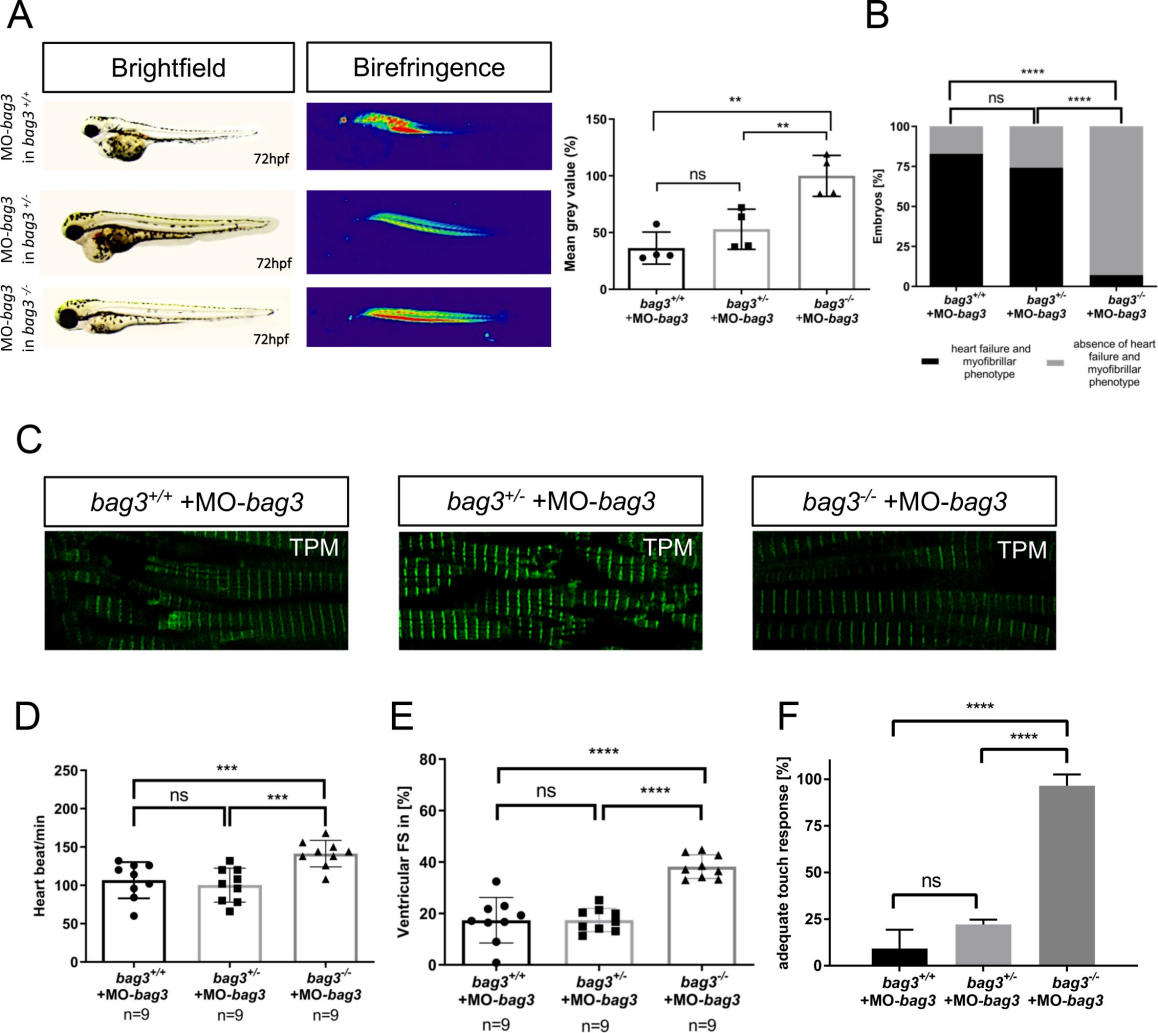
**Knockdown of Bag3 leads to heart and skeletal muscle dysfunctions only in *bag***^***+/+***^ ***and bag3***^***+/-***^ **embryos. (A)** Brightfield and birefringence images of *bag3*^*+/+*^, *bag3*^*+/-*^ and *bag3*^*-/-*^ embryos at 72 hpf injected with MO-*bag3*. Densitometric analysis of birefringence (n = 4). Representative samples are shown (One-way ANOVA followed by tukey's multiple comparison analysis, P = 0.0012). **(B)** Only *bag3*^*-/-*^ embryos injected with MO-*bag3* do not show (cardio-)myopathy, whereas *bag3*^*+/+*^and *bag3*^*+/-*^ develop the characteristic *bag3* morphant phenotype (N = 3, n = 40, mean ± S.D., P<0.0001, two-tailed value for Fisher´s exact test. *bag3*^*+/+*^ and *bag3*^*+/-*^ P = 0.5346*; bag3*^*+/+*^ and *bag3*^*-/-*^ P<0.0001; *bag3*^*+/-*^ and *bag3*^*-/-*^ P<0.0001). **(C)** Tropomyosin immunostainings of *bag3*^*+/+*^, *bag3*^*+/-*^, and *bag3*^*-/-*^ embryos injected with MO-*bag3* at 72 hpf reveal that only *bag3*^*-/-*^ mutant embryos injected with MO-*bag3* does not develop muscle fiber disruptions. **(D)** Heart rate quantification at 72 hpf reveals impairments only in *bag3*^*+/+*^and *bag3*^*+/-*^ embryos injected with MO-*bag3* (N = 3, n = 9, mean ± S.D. One-way ANOVA followed by tukey's multiple comparison analysis, P = 0.0008). **(E)** Quantification of ventricular FS at 72 hpf reveals contractile dysfunctions only in *bag3*^*+/+*^and *bag3*^*+/-*^ embryos injected with MO-*bag3* (N = 3, n = 9, mean ± S.D. One-way ANOVA followed by tukey's multiple comparison analysis, P<0.0001). **(F)** *bag3*^*-/-*^ embryos injected with MO-*bag3* show proper flight response upon mechanical stimulus, whereas *bag3*^*+/+*^and *bag3*^*+/-*^ embryos injected with MO-*bag3* do not show an adequate touch response (N = 3, n = 60, mean ± S.D. One-way ANOVA followed by tukey's multiple comparison analysis, P<0.0001).

To validate whether these findings were specifically caused by the loss of Bag3 or were due to off-target effects of the morpholino, we injected the same concentration of the *bag3*-specific morpholino (MO-*bag3*) into mutant *bag3*^*-/-*^ embryos as well as clutchmate controls (*bag3*^*+/-*^ and *bag3*^*+/+*^ embryos). Next, we screened these embryos for specific *bag3*-associated phenotypic alterations. Similar to MO-*bag3*-injected wild-type embryos (N = 3, n = 140/160 P<0.0001) (Fig 3A), *bag3*^*+/-*^ and *bag3*^*+/+*^ embryos injected with MO-*bag3* showed severe pericardial edema and muscle fiber disorganization as well as heart and skeletal muscle dysfunction (N = 3, n = 40 P<0.0001) (Fig 4A–4F). By contrast, injection of MO-*bag3* into homozygous mutant *bag3*^*-/-*^ embryos did not cause cardiomyopathy and skeletal muscle dysfunction (Fig 4A–4F). Specifically, muscle structure (evaluated by Tropomyosin immunostainings) in MO-*bag3-*injected *bag3*^*+/-*^ and *bag3*^*+/+*^ embryos was indistinguishable from wild-type embryos injected with MO-*bag3* (Fig 4A and 4C). By contrast, muscle structure in *bag3*^*-/-*^ embryos injected with MO-*bag3* is well preserved and indistinguishable from wild-type muscle structure (Fig 4A–4C). Moreover, *bag3*^*-/-*^ embryos injected with MO-*bag3* did not develop functional heart and skeletal muscle defects compared to MO-*bag3*-injected *bag3*^*+/+*^ and *bag3*^*+/-*^ embryos as indicated by the assessment of the heart rate (N = 3, n = 9, P = 0.0008. HR *bag3*^*+/+*^: 106±23.58 heart beat/min; HR *bag3*^*+/-*^: 100 ±22.21 heart beat/min; HR *bag3*^*-/-*^: 141±17.26 heart beat/min, at 72 hpf), contractile force by FS measurements (N = 3, n = 9, P<0.0001. FS *bag3*^*+/*+^: 17.38±8.84%; FS *bag3*^*+/-*^: 17.44±4.54%; FS *bag3*^*-/-*^: 38.23±4.59%, at 72 hpf), and touch evoked assays (N = 3, n = 60, P<0.0001. Mot *bag3*^*+/+*^: 9.23±5.69%; Mot *bag3*^*+/-*^: 22.14±2.25%; Mot *bag3*^*-/-*^: 96.49±3.18%, at 72 hpf) (Fig 4D–4F; S2, S4, S9 and S10 Movies).

These findings clearly demonstrate the specificity of the morpholino-mediated Bag3 knockdown and indicate that CRISPR/Cas9-induced Bag3 ablation might trigger genetic compensation to prevent (cardio-)myopathy.

### Bag2 mediated genetic compensation triggered by CRISPR/Cas9-induced Bag3 deletion prevents heart and skeletal muscle dysfunction

**Fig 5:**
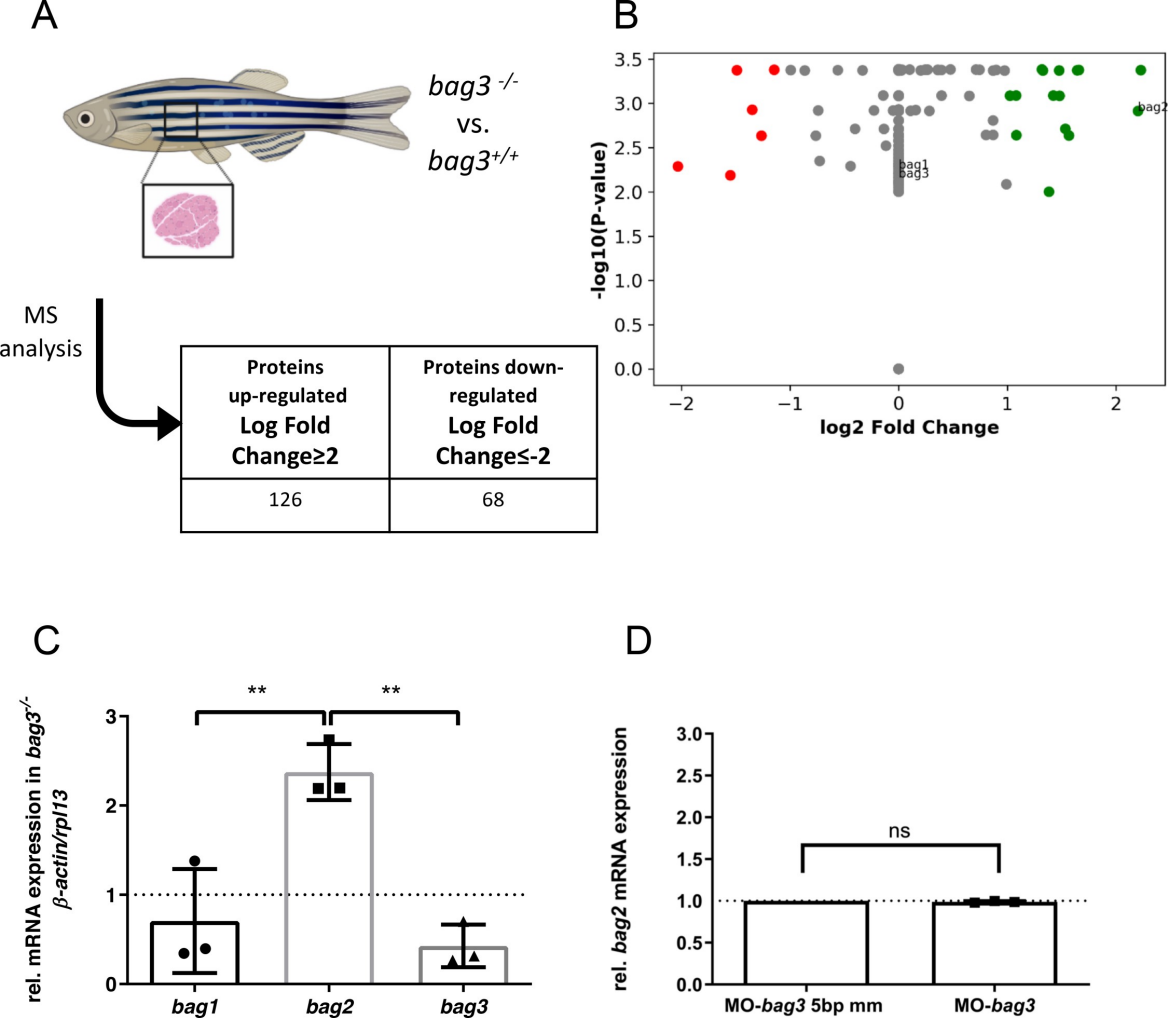
Bag2 mediates genetic compensation in *bag3* knockout zebrafish. **(A)** Schematic description of proteomic workflow. Acquired spectra were analyzed against the Uniprot zebrafish database using MaxQuant. **(B)** Detection levels of label free quantification (LFQ) intensity ratio between *bag3*^*-/-*^ and *bag3*^*+/+*^ zebrafish samples of three orthologs of the Bag family protein, Bag1, Bag2 and Bag3. The values are shown as Log2 fold change ≥2 and ≤2. **(C)** Quantitative real-time PCR of *bag3*^*-/-*^ and *bag3*^*+/+*^ zebrafish embryos at 72 hpf shows significant upregulation of *bag2* mRNA levels, whereas *bag1* transcript levels are not increased (N = 3, mean±S.D, One-way ANOVA followed by tukey's multiple comparison analysis **bag1 vs *bag2* P = 0.0057, ** *bag2* vs *bag3* P = 0.0026). **(D)** Quantitative real-time PCR of MO-*bag3*- or MO-*bag3* 5bp mm-injected embryos shows no upregulation of *bag2* mRNA levels in *bag3* morphants at 72 hpf. (n = 3, mean±S.D, P = 0.1080 determined using two-tailed t-test).

Next, to assess whether genetic compensation is activated in our CRISPR/Cas9-induced *bag3* mutants, we performed a mass spectrometry (MS)-based proteomic analysis on muscle tissue derived from adult homozygous *bag3*^*-/-*^ mutants compared to homozygous wild-type *bag3*^*+/+*^ clutchmates (Fig 5A). The obtained proteomics profiles revealed 68 down- and 126 up-regulated proteins (log fold change >2 and <-2) (Fig 5B; S2 Table). As expected, Bag3 was not detected in homozygous *bag3*^*-/-*^ mutant muscle tissue (Fig 5B), confirming Bag3 deficiency in muscle tissue of our CRISPR/Cas9-induced mutant line. Interestingly, among the most up-regulated proteins, we found levels of Bag2, a member of the Bag protein family, significantly elevated (Log Fold Change = 2.2). This suggested Bag2 could be a potential functional compensator for the loss of Bag3.

Next, to validate transcriptional adaptation/upregulation of Bag2 in *bag3*^*-/-*^ mutants, we performed quantitative real-time PCR analyses of *bag1*, *bag2*, and *bag3*. Initially, we confirmed that the three *bag* family members, *bag1-3*, are expressed in wild-type zebrafish during development and in the adult skeletal muscle (S4A–S4D Fig). In *bag3*^*-/-*^ mutant embryos and adult skeletal muscle, *bag1* mRNA levels were transcriptionally unaltered compared to wild-types, whereas *bag3* mRNA levels were significantly reduced (N = 3, **P = 0.0071, ***P = 0.0007) (Fig 5C; S4E Fig), confirming *bag3* mutant mRNA decay. In accordance with the proteomic analyses, *bag2* transcript levels were found to be significantly upregulated in *bag3*^*-/-*^ mutant embryos and adult zebrafish skeletal muscle (Fig 5C; S4E Fig). By contrast, *bag2* transcript levels were not found to be upregulated in zebrafish embryos injected with *bag3*-specific morpholinos (MO-*bag3*) (Fig 5D).

**Fig 6:**
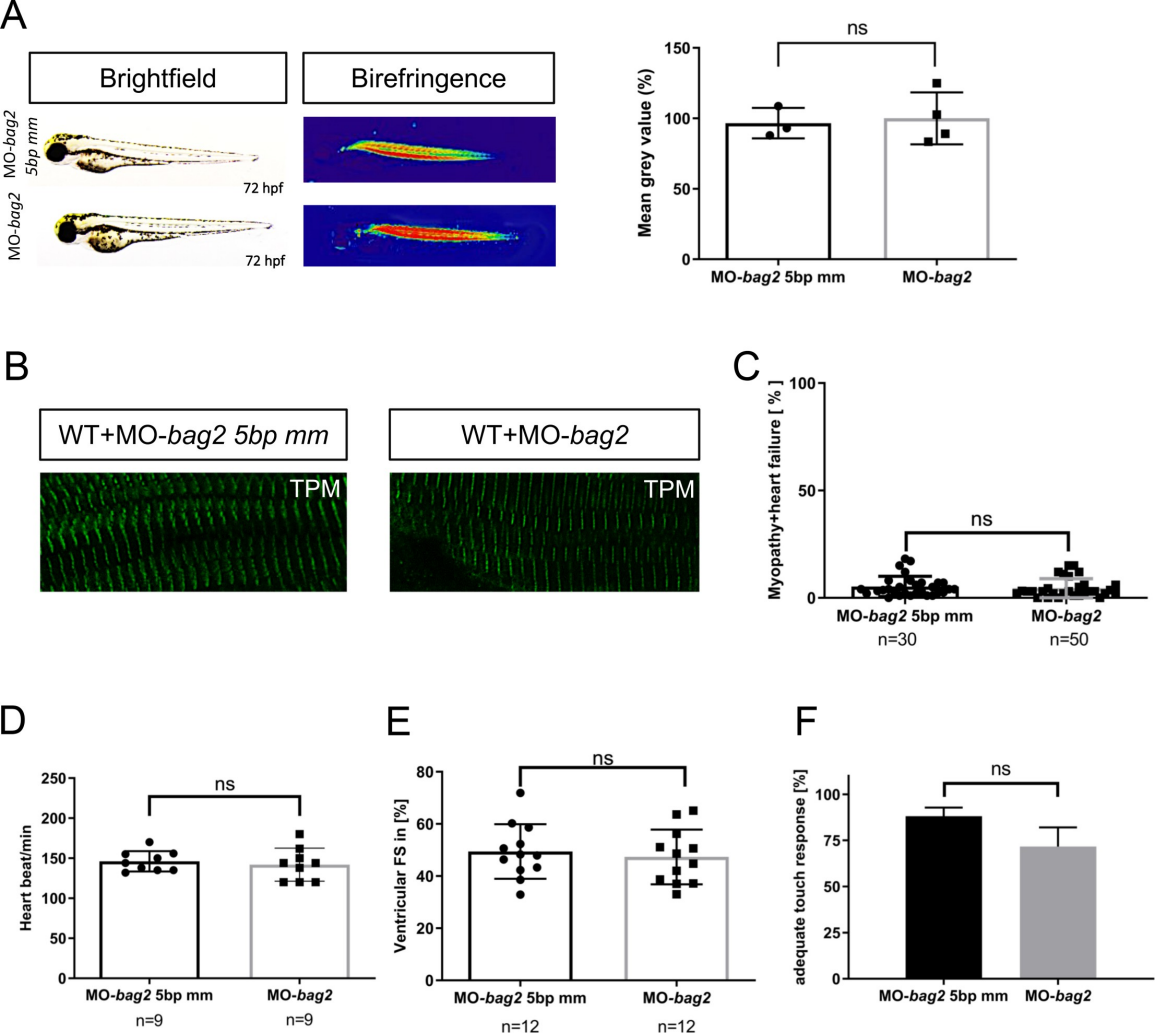
Targeted knockdown of *bag2* does not lead to heart and muscles dysfunction in zebrafish embryos. **(A)** Brightfield and birefringence images and the densitometric analysis of birefringence (n = 4) of MO-*bag2* and MO-*bag2* 5bp mm injected embryos reveal that *bag2* morphants do not develop a (cardio)-myopathy phenotype by 72 hpf. Representative samples are shown (P = 0.7891 determined using two tailed t-test). **(B)** Tropomyosin immunostainings of MO-*bag2-* and MO-*bag2* 5bp mm-injected embryos at 72 hpf demonstrate that *bag2* morphants do not develop fiber disruptions. **(C)** *bag2* morphants do not develop (cardio-)myopathy (only 4.50±4.43% of embryos exhibit a phenotype) (N = 3, n = 30/50, Mean± SD P = 0.5010 determined using two-tailed t-tests). **(D)** Heart rate quantification of *bag2* morphants (146±12.64 heart beat/min), at 72 hpf does not show any impairment compared to *bag2* 5bp-mismatch morphants (142±20.57 heart beat/min) (N = 3, n = 9, mean ± S.D. P = 0.6490 determined using two-tailed t-tests). **(E)** FS of *bag2* morphant ventricles at 72 hpf is not reduced (47.34±10.49%) compared to 5bp-mismatch-MO injected embryos (FS: 49.44±10.48%) (N = 3, n = 12, Mean± SD P = 0.6279 determined using two-tailed t-tests). **(F)** Touch evoked assay for *bag2* morphants does not reveal a significant reduction in responsiveness to mechanical stimulus (71.67±4.22%) (N = 3, n = 50/25, mean ± S.D. P = 0.0941, determined using two-tailed t-tests).

Bag2 (Bcl2-associated athanogene 2) is a molecular co-chaperone and a member of the Bag protein family that shows high structural similarity to Bag3. In zebrafish, Bag2 was found to be expressed in the heart and skeletal muscle [19]. To first investigate the role of Bag2 *in vivo*, we inactivated zebrafish *bag2* by injecting a morpholino against the splice donor site of exon 2 (MO-*bag2*) into one-cell stage zebrafish embryos. When injected with either MO-*bag2* or the 5bp mismatch control morpholino (MO-*bag2* 5bp mm), injected embryos (N = 3, n = 30/50, P<0.5010) did not develop (cardio-)myopathy (Fig 6A–6F)). We found normal cardiac and skeletal muscle structure and function (Fig 6A and 6B), unconfined cardiac contractile function (Fig 6D and 6E), and undisturbed motility (Fig 6F) in *bag2* morphant embryos. This demonstrates that Bag2 is dispensable for early heart and skeletal muscle development and function.

**Fig 7:**
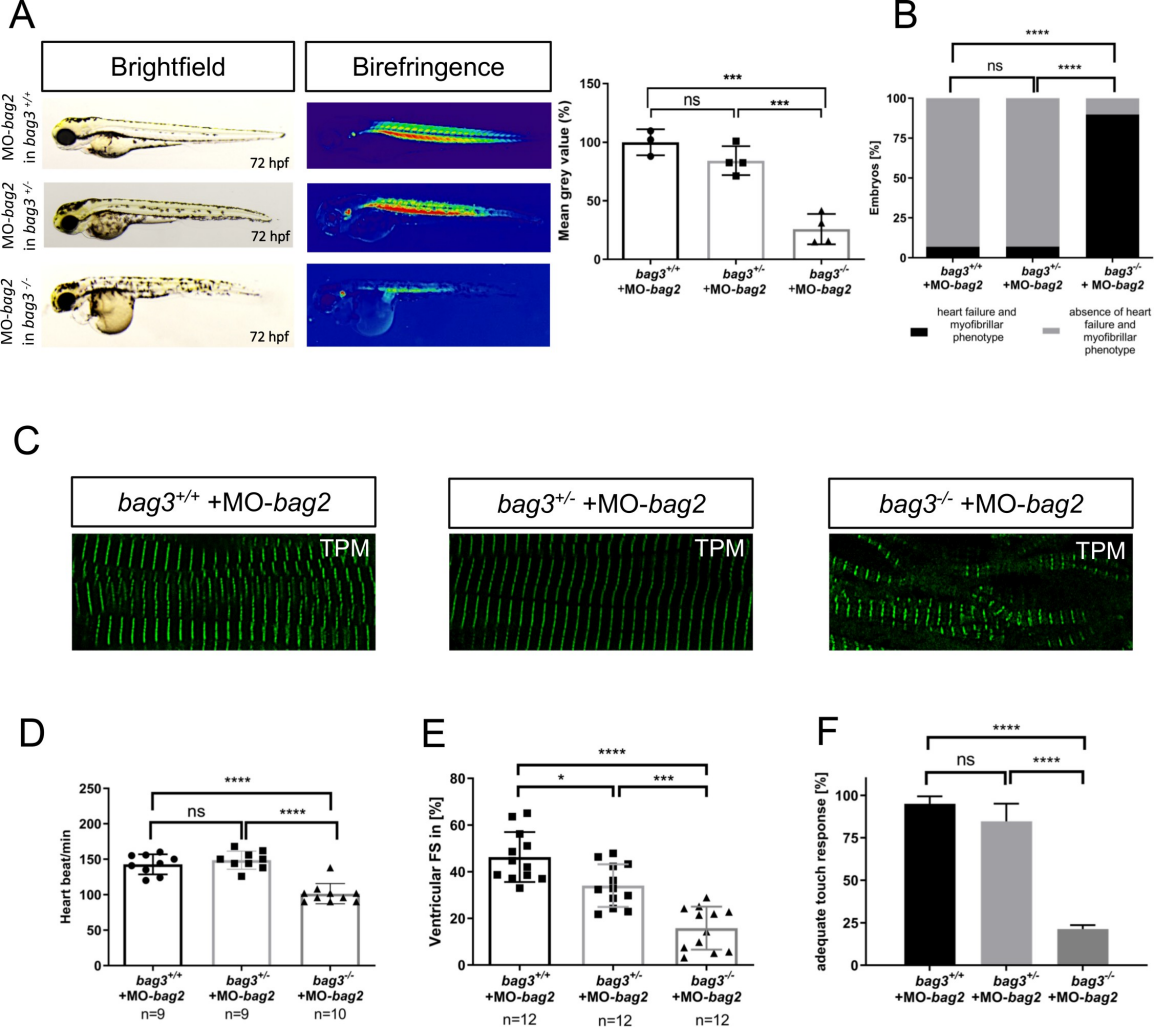
Knockdown of *bag2* in *bag3*^*-/-*^ mutants causes heart and skeletal muscle disruptions. **(A)** Brightfield and birefringence images and densitometric analysis of birefringence (n = 4) of *bag3*^*+/+*^, *bag3*^*+/-*^ and *bag3*^*-/-*^ embryos at 72 hpf injected with 200μM of MO-*bag2*. Representative samples are shown (One-way ANOVA followed by tukey's multiple comparison analysis, P<0.0004). **(B)** *bag3*^*-/-*^ embryos injected with MO-*bag2* develop (cardio-)myopathy (89.76±4.37%), whereas *bag3*^*+/+*^ (93.10±0.33%) and *bag3*^*+/-*^ (94.77±2.35%) embryos are unaffected by MO-*bag2* injection (N = 3, n = 50, mean ± S.D., P<0.0001, two-tailed value for Fisher´s exact test). **(C)** Tropomyosin immunostainings of *bag3*^*+/+*^, *bag3*^*+/-*^ and *bag3*^*-/-*^ embryos injected with MO-*bag2* at 72 hpf. Only MO-*bag2*-injected *bag3*^*-/-*^ embryos show muscle fiber disruptions. **(D)** Heart rate quantification at 72 hpf reveals impairments only in *bag3*^*-/-*^ embryos injected with MO-*bag2* (N = 3, n = 9/10, mean ± S.D. One-way ANOVA followed by tukey's multiple comparison analysis, P<0.0001). **(E)** FS of ventricles of *bag2* splice MO injected in *bag3*^*-/-*^ embryos at 72 hpf (FS: 15.83±9.19%) is significantly reduced compared to the FS measured in *bag3*^*+/+*^ (FS: 46.33±10.72%) and *bag3*^*+/-*^ (FS: 34.07±9.17%) injected embryos (N = 3, n = 12; mean± SD One-way ANOVA followed by tukey's multiple comparison analysis, *P = 0.0110,***P = 0.0002, ****P< 0.0001). **(F)** *bag3*^*-/-*^ embryos injected with MO-*bag2* reveal significantly impaired responsiveness upon mechanical stimulus compared to *bag3*^*+/+*^and *bag3*^*+/-*^ embryos injected with MO-*bag2* (N = 3, n = 80/50, mean ± S.D. One-way ANOVA followed by tukey's multiple comparison analysis, P<0.0001).

Next, to investigate the role of Bag2 as putative compensatory factor in the CRISPR/Cas9-induced Bag3 knockout line, we inactivated *bag2* by injecting MO-*bag2* into *bag3*^*+/+*^, *bag3*^*+/-*^, and *bag3*^*-/-*^ embryos. While Bag2 knockdown in *bag3*^*+/+*^ and *bag3*^*+/-*^ embryos did not result in heart or skeletal muscle malformation and dysfunction (Fig 7A–7F), 89.76 ± 4.37% of *bag3*^*-/-*^ mutant embryos injected with MO-*bag2* developed severe heart and skeletal muscle defects comparable to the phenotypic characteristics observed in *bag3* morphants (N = 3, n = 50, P<0.0001) (Fig 7A–7F). In detail, we found that MO-*bag2* injection into *bag3*^*-/-*^ embryos caused severe disruption of muscle structure and ultrastructure (Fig 7C; S3A Fig), whereas sarcomeric organization in MO-*bag2*-injected *bag3*^*+/+*^ and *bag3*^*+/-*^ embryos was completely unaffected (Fig 7C). Furthermore, *bag3*^*-/-*^ embryos injected with MO-*bag2* showed severe functional heart and skeletal muscle defects compared to MO-*bag2*-injected *bag3*^*+/+*^ and *bag3*^*+/-*^ embryos as assessed by the measurement of the heart rate (N = 3, n = 9/10, P<0.0001. HR *bag3*^*+/+*^: 143±14.18 heart beat/min; HR *bag3*^*+/-*^: 148±12.65 heart beat/min; HR *bag3*^*-/-*^: 101±14.27 heart beat/min, at 72 hpf), contractile force (N = 3, n = 12, P<0.0001. FS *bag3*^*+/*+^: 46.33±10.72%; FS *bag3*^*+/-*^: 34.07±9.17%; FS *bag3*^*-/-*^: 15.83±9.19%, at 72 hpf), and touch evoked assays (N = 3, n = 80/50, P<0.0001. Mot *bag3*^*+/+*^: 95±4.41%; Mot *bag3*^*+/-*^: 84.68±10.38%; Mot *bag3*^*-/-*^: 21.33±2.31%, at 72 hpf) (Fig 7D–7F; S3, S5 and S11 Movies). By contrast, specification of atrial and ventricular chambers was not affected by the knockdown of *bag2* in *bag3*^*-/-*^ embryos (S3B Fig). Efficiency of the *bag2* knockdown was demonstrated by RT-PCR-based mRNA splicing assays (S6B Fig).

Similar experiments as described for *bag2* were conducted for *bag1*, but no phenotypic alterations could be recognized neither after injection of *bag1*-specific morpholinos into wild-type embryos nor *bag3*^*-/-*^ embryos. This further supports the hypothesis that *bag2* and not *bag1* effectively compensates for the loss of Bag3 in *bag3*^*-/-*^ embryos (S5A–S5E Fig, S6B and S6C Fig and S13 and S14 Movies).

### Blockade of *bag3* mRNA decay inhibits transcriptional adaptation and induces cardiomyopathy and skeletal muscle dysfunction in Bag3-deficient zebrafish

**Fig 8:**
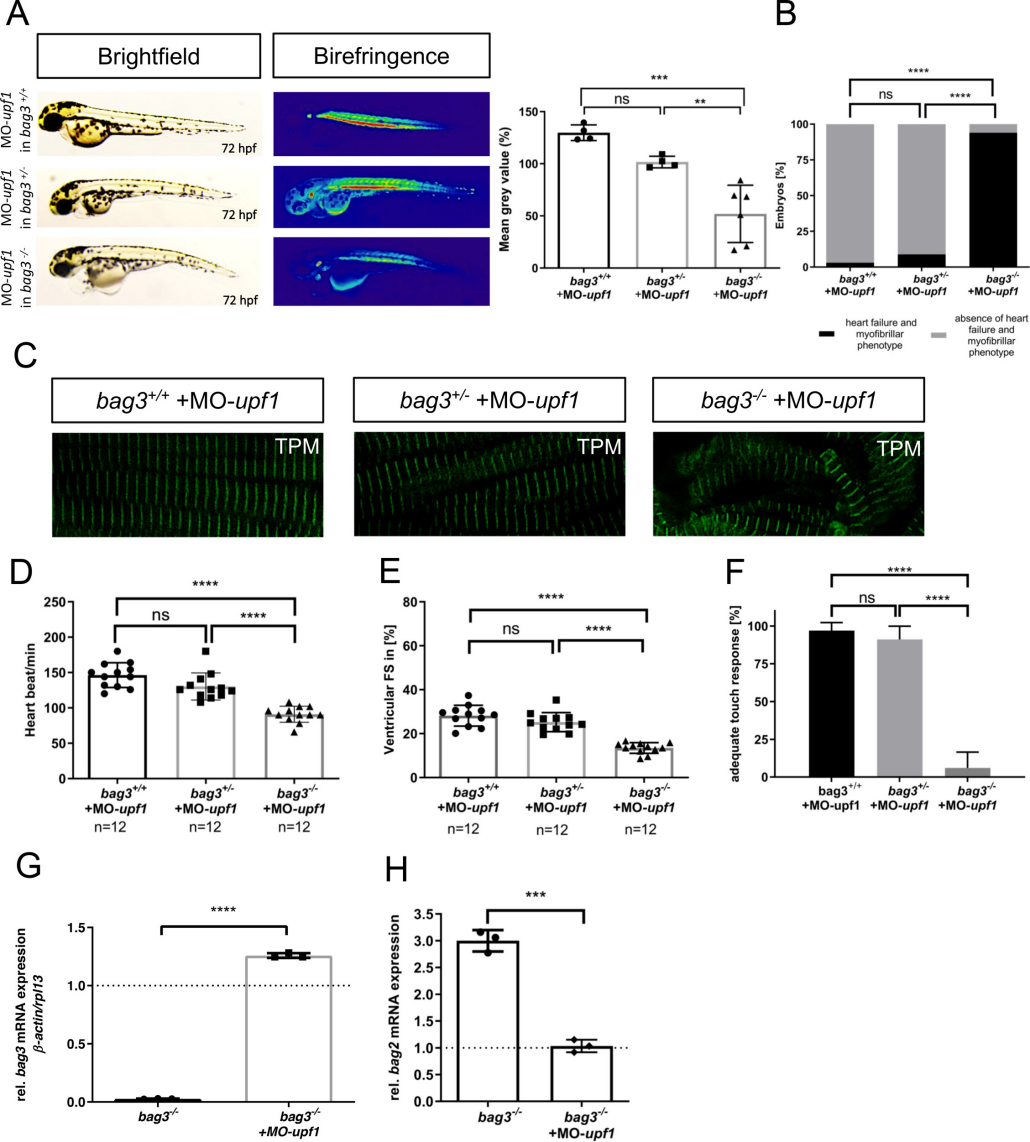
Knockdown of *upf1* blocks mutant mRNA decay required for transcriptional adaptation in *bag3^-/-^* mutants. **(A)** Brightfield and birefringence images and densitometric analysis of birefringence (n = 4/6) of *bag3*^*+/+*^, *bag3*^*+/-*^ and *bag3*^*-/-*^ embryos at 72 hpf injected with 50μM MO-*upf1*. Individual samples are shown (One-way ANOVA followed by tukey's multiple comparison analysis, **P=0.0053, ***P=0.0002). **(B)** *bag3*^*-/-*^ embryos injected with MO-*upf1* develop (cardio-)myopathy (93.94±10.50%), whereas *bag3*^*+/+*^ (96.97±5.25%) and *bag3*^*+/-*^ (91.09±8.82%) embryos are devoid of any phenotype (N = 3, n = 40, mean ± S.D., P<0.0001, two-tailed value for Fisher´s exact test). **(C)** Tropomyosin immunostainings of *bag3*^*+/+*^, *bag3*^*+/-*^ and *bag3*^*-/-*^ embryos injected with MO-*upf1* at 72 hpf reveal muscle fiber disruptions due to the blockage of the transcriptional adaptation only in *bag3*^*-/-*^ injected embryos. **(D)** Heart rate quantification at 72 hpf reveals impairments only in *bag3*^*-/-*^ embryos injected with MO-*upf1* (N = 3, n = 12, HR *bag3*^*+/+*^: 146±17.49 heart beat/min; HR *bag3*^*+/-*^: 130±19.18 heart beat/min; HR *bag3*^*-/-*^: 91±11.39 heart beat/min, mean ± S.D. One-way ANOVA followed by tukey's multiple comparison analysis, P<0.0001). **(E)** FS of ventricles of *bag3*^-/-^ embryos injected with MO-*upf1* is significantly reduced at 72 hpf (FS: 13.48±2.438%) compared to the FS measured in *bag3*^*+/+*^ (FS: 28.17±4.753%) and *bag3*^*+/-*^ (FS: 25.23±4.328%) morphants (N = 3, n = 12; mean± SD One-way ANOVA followed by tukey's multiple comparison analysis, P<0.0001). **(F)** *bag3*^*-/-*^ embryos injected with MO-*upf1* (6.06±10.5%) reveal significant difference in responsiveness upon mechanical stimulus compared to *bag3*^*+/+*^(96.97±5.25%) and *bag3*^*+/-*^ (91.09±8.82%) injected embryos (N = 3, n = 35, mean ± S.D. One-way ANOVA followed by tukey's multiple comparison analysis, P<0.0001). **(G)** Quantitative real-time PCR of *bag3*^*-/-*^ +MO-*upf1* and *bag3*^*-/-*^ zebrafish embryos at 72 hpf shows restored *bag3* mRNA levels only in *bag3*^*-/-*^ embryos injected with MO-*upf1* (N = 3, mean ± SD, P<0.0001 determined using two-tailed t-test). **(H)** Quantitative real-time PCR of *bag3*^*-/-*^ embryos injected with MO-*upf1*and uninjected *bag3*^*-/-*^ control embryos at 72 hpf reveals the downregulation of *bag2* mRNA levels only in *bag3*^*-/-*^ + MO-*upf1* embryos (N = 3, mean ± SD, P = 0.0004 determined using two-tailed t-test).

To investigate whether the inhibition of nonsense-mediated decay (NMD) of mRNA and the subsequent blockade of the transcriptional adaptation machinery [1–3] can induce heart and skeletal muscle defects in *bag3*^*-/-*^ embryos, we inactivated *upf1*, a factor known to be crucial for regular NMD function[20, 21]. We found that morpholino-mediated knockdown of *upf1* (MO-*upf1*) in *bag3*^*+/+*^ and *bag3*^*+/-*^ embryos did not result in heart and skeletal muscle defects, whereas knockdown of *upf1* in *bag3*^*-/-*^ embryos led to severe structural and functional heart and skeletal muscle defects (Fig 8A–8F). Muscle structure was disrupted in *bag3*^*-/-*^ embryos injected with MO-*upf1* (Fig 8C), while sarcomeric organization in MO-*upf1*-injected *bag3*^*+/+*^ and *bag3*^*+/-*^ embryos was preserved (Fig 8C). Furthermore, MO-*upf1* injection into *bag3*^*-/-*^ embryos caused severe functional heart and skeletal muscle defects, however, MO-*upf1*-injected *bag3*^*+/+*^ and *bag3*^*+/-*^ embryos showed no phenotypic alterations (Fig 8D–8F; S6, S7 and S12 Movies). High efficiency of the morpholino-mediated *upf1* knockdown was confirmed by RT-PCR-based mRNA splicing assays (S6A Fig).

Finally, to assess whether inhibiting NMD in *bag3*^*-/-*^ embryos indeed resulted in the preservation of *bag3* mRNA levels and reduction of *bag2* mRNA levels, we performed qRT-PCR analyses in MO-*upf1*-injected *bag3*^*-/-*^ embryos. Indeed, we found the preservation of mutant *bag3* mRNA levels as well as the diminution of *bag2* mRNA levels to physiological levels in *bag3*^*-/-*^ embryos injected with MO-*upf1*, (Fig 8G and 8H), indicating that blocking the NMD of *bag3* mRNA effectively inhibits the transcriptional adaptation of *bag2* expression.

In summary, our findings demonstrate that transcriptional adaptation of *bag2* expression in a model of CRISPR/Cas9-induced Bag3 ablation is involved in the process of genetic compensation to preserve heart and skeletal muscle structure and function *in vivo*.

## Discussion

Genetic compensation by transcriptional adaptation was recently described to contribute to genetic robustness that guarantees viability and fitness of an organism in the presence of malignant and harmful gene variations and mutations [1, 2]. Here, we describe compensatory transcriptional adaptation in a CRISPR/Cas9-induced zebrafish model of Bag3-deficiency. Whereas morpholino-mediated ablation of Bag3 in zebrafish embryos led to severe heart failure and myopathic phenotypes, the knockout of Bag3 by CRISPR/Cas9 technology was not accompanied by phenotypic alterations in the developing embryo. We found that in our CRISPR/Cas9-induced *bag3* mutant embryos, the expression of the Bag protein family member Bag2 was significantly upregulated.

El-Brolosy et al. proposed an explanatory model for the discrepancy in transcriptional adaptation in mutants and morphants. It was found that degradation of the mutated mRNA by mRNA degradation pathways is the molecular trigger for the upregulation of genes that exhibit sequence similarity thereby enabling functional compensation [1, 22, 23]. Pharmacological but also genetic inhibition of NMD (e.g. by the inactivation of Upf1, a factor pivotal for NMD) led to a significant reduction of mutated mRNA and the loss compensatory transcriptional adaptation [2, 23, 24].

BAG3 is a molecular co-chaperone that interacts with heat shock proteins to facilitate important functions in protein homeostasis such as autophagy [5, 6]. Interestingly, BAG3 is predominantly expressed in the heart and skeletal muscle cells and mutations in the *BAG3* gene are associated with different variants of cardiomyopathies such as hypertrophic cardiomyopathy (HCM) and DCM or protein aggregation diseases such as MFM [8–10, 15]. In 2011, two independent genome-wide association studies (GWAS) linked mutations in the human BAG3 gene to DCM [9, 12]. Based on these initial findings, human genetic studies confirmed these results and introduced BAG3 as a major DCM causing gene with an incidence of up to 6.7% of all DCM cases [17, 25, 26]. To date, several different human BAG3 mutations are described to be associated with DCM. Remarkably, DCM-associated BAG3 mutations are frequently found to result in the truncation of the BAG3 protein and are assumed to cause the complete loss of BAG3 function [25, 27]. These findings are supported by analyses in conditional, cardiomyocyte-specific Bag3 knockout mice that also develop DCM shortly after birth [28]. To the best of our knowledge, DCM-associated truncating human BAG3 mutations, predominately frame shift and nonsense mutations [25], were never analyzed for the induction of the NMD of the mutated *bag3* mRNA and thereby the activation of putative compensatory mechanisms.

Global disruption of the Bag3 gene in homozygous mice led to lethality after 4 weeks of age. These BAG3 knockout mice were characterized by severe myopathic phenotypes including disruption of Z-disk architecture and myofibrillar degeneration [15]. Cardiac-specific knockout of BAG3 by the use of cardiomyocyte-specific α-myosin heavy chain Cre-transgenic mice (αMHC-Cre mice) significantly impaired structure and function of the contractile apparatus in cardiomyocytes resulting in DCM and premature death of these conditional Bag3 knockout mice during their first year of life [28]. Fang and co-workers did not observe increased expression of other BAG family members such as Bag 1 or Bag2 in their conditional Bag3 knockout model [28], implying that their knockout strategy might not activate NMD-related genetic compensation. Knockdown of Bag3 in the zebrafish model using morpholino-modified antisense oligonucleotides resulted in the development of cardiac contractile dysfunction and cardiomyopathy [9] as well as a contraction-dependent myofibrillar disintegration and skeletal muscle dysfunction, two of the characteristics of BAG3-related myofibrillar myopathies (MFM) [10, 11]. Very recently, Ding et al. described that TALEN-mediated ablation of Bag3 led to cardiomyopathy in adult zebrafish most likely due to interference with protein degradation and homeostasis [17].

Here, we describe a CRISPR/Cas9-induced zebrafish model of Bag3 deficiency and we show that *bag3* mutant zebrafish embryos, in contrast to *bag3* morphants, do not develop heart and skeletal muscle defects. Expression of Rpl10, the 60S ribosomal protein L10 and Bag2 were found to be strongly induced in *bag3*^*-/-*^ embryos. Loss of Rpl10 was shown to cause neurodevelopmental defects in zebrafish [29], but does not show any sequence or domain similarities to Bag3 making Rpl10 unlikely to functionally compensate for the loss of Bag3. By contrast, Bag2, a member of the Bag protein family, shows high sequence and domain similarities to Bag3 turning Bag2 into a perfect candidate to compensate for the loss of Bag3 function. In humans, BAG2 is expressed in several tissues, including brown adipose and the lung but also the heart [30]. Unfortunately, not much is known about the *in vivo* function of Bag2. To date, no *bag2*-null animal models are described. Here, we knocked down Bag2 by morpholinos in the zebrafish embryo and found no phenotypic alterations in the morphants. By contrast, injection of MO-*bag2* into homozygous mutant *bag3*^*-/-*^ zebrafish embryos led to severe heart and skeletal muscle defects, resembling the phenotypic alterations observed in *bag3* morphants. This suggests that Bag2 functionally compensates for the loss of Bag3 in our genetic model of Bag3 deficiency. El-Brolosy and colleagues found that blocking NMD in *hbegfa*^*Δ7*^, *vegfaa and vcla*^*Δ13*^ zebrafish mutants by genetically inactivating Upf1 led to the preservation of the mutated mRNA levels and, subsequently, to the loss of transcriptional adaptation [1]. Similar to these studies, we found that NMD of the mutated *bag3* mRNA triggers the transcriptional adaptation of *bag2* expression since blocking NMD by the injection of *upf1*-specific morpholinos was able to preserve mutant *bag3* mRNA levels and to reduce the *bag2* mRNA levels to normal levels in homozygous mutant *bag3*^*-/-*^ embryos. These findings imply that *bag3* mRNA degradation is the main trigger of transcriptional adaptation in *bag3*^*-/-*^ zebrafish embryos. Interestingly, *bag3*^*-/-*^ zebrafish display increased mortality starting from 9 month of age. We found that *bag2* mRNA expression was still increased in the adult skeletal muscle in *bag3*^*-/-*^ zebrafish, suggesting that transcriptional adaptation of *bag2* expression is able to compensate for the loss of *bag3* during embryogenesis but not in the adult zebrafish. Whether this is due to insufficient compensatory *bag2* levels or specific Bag2-independent molecular pathomechanisms in the adult zebrafish is unknown but should be investigated in future studies.

Recent findings from zebrafish and mouse studies suggest that genetic compensation is a widespread phenomenon which plays a vital role in the phenotypic peculiarity in response to malignant and harmful gene mutations. Human genetics studies also suggest that genetic compensation might be an active mechanism to influence clinical disease manifestation in response to severely mutated alleles [31–33]. Further elucidation of the molecular mechanisms that control genetic compensation might pave the way to develop therapeutic interventions to foster genetic robustness to a malignant mutation.

## Materials and methods

### Zebrafish strains and injection procedures

The present study was performed after appropriate institutional approvals (Tierforschungszentrum (TFZ) Ulm University, No. 0183), which conform to EU Directive 2010/63/EU. Care and breeding of zebrafish, *Danio rerio*, was conducted as described previously [34, 35]

Morpholino-modified antisense oligonucleotides (MOs; Gene Tools, LLC, Oregon, USA) were directed against the splice-acceptor/donor site of zebrafish *bag3*, *bag2*, *bag1* and *upf1*. As negative controls 5 bp mismatch MOs were injected at the same concentration as the respective MO. All MOs were resolved in 0.2 M KCl to a final amount of 200 μM MO-*bag3* and MO-*bag3* 5bp mismatch; 200μM MO-*bag2* and MO-*bag2* 5bp mismatch; 50μM MO-*upf1* and MO-*upf1* 5bp mismatch; 200μM MO-*bag1* and MO-*bag1* 5bp mismatch. Injections were performed into one-cell stage zebrafish embryos.

For CRISPR/Cas9 injections, 400 ng/μl recombinant Cas9 protein (Eupheria GmbH, Germany) was mixed with synthetic tracrRNA (100 ng/μl) and a gene-specific crRNA (Eurofins Genomics, Germany) against *bag3* (50 ng/μl) in 200 mM KCl. Sequences of CRISPR RNA oligonucleotides and Morpholinos are summarized in **S1 Table**.

### RNA extraction and quantitative real-time PCR

For RNA extraction, 30 wild-type embryos and 30 *bag3*^*-/-*^ embryos were collected at 72 hpf. To extract the RNA the RNeasy® Mini Kit was used (Qiagen) according to the manufacturer's instructions. Reverse transcription was performed by using SuperScript® III Reverse Transcriptase (Life Technologies), 1μg total RNA and oligo(dT) primer. Quantitative real-time PCR was carried out according to standard protocols using SYBR-Green master mix (Roche) and a Roche LightCycler 480 II. cDNA was generated as described above from 72 hpf embryos. To correct for sample to sample variation, housekeeping genes β-actin and rpl13 were used for normalization [36]. Sequences of primers used are summarized in **S1 Table**.

### Immunoblotting

For immunoblot analysis, from 72 hpf wild-type and *bag3*^*-/-*^ embryos, proteins lysates were prepared. 30μg of protein lysate was boiled in 5x Laemmli Buffer, separated on an SDS-PAGE (8–16%), and blotted onto polyvinylidene difluoride (PVDF) membranes. Then, the membranes were blocked using 5% milk powder in TBST for 1h at RT and incubated with primary antibodies overnight at 4°C. The membranes were washed and incubated with polyclonal anti-rabbit-IgG antibody conjugated to horseradish peroxidase. All membranes were developed using the Pierce ECL Western Blotting Substrate (Thermo Scientific) and a luminescent image analyzer (Image Quant Las4000 mini) [36].

The Bag3 antibody (1:1000 in 5% BSA) was designed against the c-terminus of zebrafish Bag3 (epitope position 213–226: LSQSSHPTREKIYR) and purchased from BioGenes GmbH, Berlin. Pan-cadherin (1:4000 in 5% milk) was purchased from Abcam (ab16505).

### Immunostaining

WT, *bag*^*+/+*^, *bag3*^*+/-*^, and *bag3*^*-/-*^ injected (MO-*bag3*, MO-*bag2* and MO-*upf1*) and non-injected embryos were euthanized with tricaine at 72 hpf. Embryos were fixed in 4% paraformaldehyde overnight at 4°C and embedded in 4% low melting agarose (melting temperature ≤65°C; Sigma) dissolved in distilled water. Longitudinal sections were cut with a Leica VT1200S vibratome to a thickness of 100μm and incubated in CAS-block solution (008120, Thermo Fisher) for 30 minutes (diluted 1:10 in distilled water). Staining was conducted in CAS-block (diluted 1:10 in distilled water). Tropomyosin staining was performed with 1:50 CH1 antibody (Hybridoma Bank) overnight at 4°C followed by 1:100 Alexa-Fluor 488 (Invitrogen, A-21121) for 2 h at RT.

Immunostaining of heart muscle myosin heavy chains MF20 (1:10, mouse monoclonal IgG2b; Hybridomabank) in co-stain with S46 (1:50, mouse monoclonal IgG1, Hybridoma Bank) was performed on Dent's fixed embryos at 72 hpf. Secondary antibodies goat anti-mouse IgG1 Alexa Fluor 488 and Alexa 555 goat ant-mouse IgG2b (both Invitrogen) were applied in dilutions of 1:100. Images were acquired using the Leica SP8 microscopes (Leica Mikrosysteme Vertrieb GmbH, Wetzlar, Germany).

### Mass spectrometry

Protein lysates derived from muscle tissue of adult homozygous *bag3*^-/-^ mutant and homozygous wild-type (*bag3*^+/+^) controls (age: 3 month) were separated using SDS-PAGE and the entire lane was subsequently processed as described previously [37] for MS analysis.

Samples were analyzed as previously described [37] by employing an U3000 RSLCnano (Thermo Fisher Scientific, Idstein, Germany) for peptide separation online coupled to an LTQ Orbitrap Velos Pro (Thermo Fisher Scientific, Bremen, Germany) mass spectrometry.

A database search was performed using MaxQuant Ver. 1.6.3.4 (www.maxquant.org) [38]. Using the built-in Andromeda search engine [39], MS/MS spectra were correlated with the UniProt zebrafish database (www.uniprot.org) for peptide identification. Carbamidomethylated cysteine was considered as a fixed modification along with oxidation (M), and acetylated protein N-termini as variable modifications. False Discovery rates were set on both, peptide and protein levels, to 0.01. For quantitation, LFQ was enabled within MaxQuant using default parameters.

### Birefringence analysis

Images were taken with an Olympus SZX 16 microscope and movies were recorded with a Leica DM IL LED microscope. The functional assessment of cardiac contractility was carried out as previously described [34, 40].

Regarding the birefringence analysis, 72 hpf zebrafish embryos were anesthetized with tricaine and embedded in 2.5% methylcellulose, in a glass petri dish, making sure that they were lying as flat as possible. Images were acquired between two polarizing filters on an Olympus SZX16 with a DP72 camera and the Olympus Stream software. After acquisition, the inverted images were analysed using the Fiji software and the mean grey value of the selected area of the skeletal muscles was measured. Data analysis was performed normalizing the mean intensity with the selected area as previously described [16, 41]. For the publication, the analysed images where optimized from black and white to colored heat map using the tool display “LUT” and selecting the “physics” option in Fiji software.

### Functional assessment

For the touch evoked assay, zebrafish embryos at 72 hpf were touched by a needle tip and their flight response was analysed. An immediate and straightforward flight was regarded as “adequate” while no movement or a delayed/incomplete response were regarded as “inadequate”. Movies of 10–30 seconds were acquired for each sample to show the larval response to the needle touch. For each experiment the number of embryos with adequate response to the touch was converted to a percentage, analysed, and plotted in a bar graph using GraphPad Prism software[11, 42].

To increase the workload on the skeletal muscles, embryos at 120 hpf were incubated in 1% methylcellulose for 2 hours as previously described [10].

### Statistical analysis

Statistical analyses were performed using GraphPad Prism software. All the experiments were conducted in triplicate using different biological samples.

All results are expressed as mean ± standard deviation (S.D.) and statistical analysis were performed as indicated in the figure legends. A p-value smaller than 0.05 was regarded as statistically significant.

## Supporting information

S1 Fig Human (hs_BAG3) and zebrafish (dr_Bag3) BAG3 show high amino acid homology particularly within functional domains (77%). Overall amino acid identity between human and zebrafish BAG3.
(PDF)

S2 Fig Kaplan–Meier survival curves of *bag3* ^*-/-*^ adult fish and adult *bag3*^*+/+*^ controls. (n = 15, log-rank test. Mean ± s.e.m. P = 0.0052). (PDF)

S3 Fig (a) Transmission electron microscopic (TEM) analysis of parasagittal and transversal sections through cardiac and skeletal muscle cells of WT, *bag3*^*-/-*^ and *bag3*^*-/-*^ + MO-*bag2* embryos at 72 hpf. In contrast to the WT and *bag3*^*-/-*^, *bag3*^*-/-*^ + MO-*bag2* cardiomyocytes and fast-twitch skeletal muscle fibers show disrupted sarcomeric structures. (b) Hearts at 72 hpf stained with chamber-specific myosin antibodies, MF20 (red) and S46 (green) (MF20 marks the entire heart and S46 is atrium specific). Heart chamber specification appears to be normal in all analyzed embyros.
(PDF)

S4 Fig Expression levels detected by qPCR of *bag1* (a), *bag2* (b) and *bag3* (c) in WT embryos at different hours post fertilization (24, 48, 72 and 120 hpf). The graph shows a very similar expression pattern for *bag1* and *bag2*. (d) qPCR showing *bag1*, *bag2* and *bag3* levels in adult zebrafish skeletal muscles (N = 3, mean±S.D, One-way ANOVA followed by tukey's multiple comparison analysis P = 0.8457). (e) *bag2* transcript levels are significantly increased in skeletal muscle of adult *bag3*^*-/-*^ zebrafish (N = 3, mean±S.D, One-way ANOVA followed by tukey's multiple comparison analysis ***P = 0.0003).
(PDF)

S5 Fig (a) Brightfield and birefringence images of *bag1* splice MO injected WT and *bag3*^*-/-*^ embryos at 72 hpf don´t show any (cardio)-myopathy phenotype. The densitometric analysis of birefringence signals supports the absence of sarcomeric disorganization. Individual samples are shown (n = 4, P>0.9999 determined using two tailed t-test). (b) MF20 and S46 immunostainings of WT and *bag3*^*-/-*^ embryos at 72 hpf injected with MO-*bag1* reveal regular specification of the cardiac chambers. (c) Heart rate quantification of *bag1* morphants and *bag3*^*-/-*^ + MO-*bag1* at 72 hpf reveals no functional cardiac impairments (N = 3, n = 9/10. HR MO-*bag1* injected WT embryos: 167±11.40 heart beat/min; HR *bag3*^*-/-*^ +MO-*bag1*: 167±9.98 heart beat/min; mean ± S.D. P = 0.9894 determined using two-tailed t-tests). (d) Ventricular FS of *bag1* morphants (20.59±8.47%) and *bag3*^*-/-*^ + MO-*bag1* embryos (FS: 22.12±4.20%) at 72 hpf is unaltered (N = 3, n = 9; Mean± SD P = 0.6373 determined using two-tailed t-tests).
(PDF)

S6 Fig (a) Injection of MO-*upf*1 (splice-blocking morpholino) results in the partial skipping of *upf*1 exon 1, leading to a frame shift, a premature stop codon and the premature termination of Upf1 translation [43, 44]. (b) Injection of MO-*bag2* (splice-blocking morpholino) results in the integration of the intron 2, a frame shift, a premature stop codon and the premature termination of Bag2 translation. (c) Injection of splice MO-*bag1* (splice-blocking morpholino) results in the skipping of the exon 2, a frame shift, a premature stop codon and the premature termination of Bag1 translation.
(PDF)

S7 Fig Additional transmission electron microscopic (TEM) pictures of parasagittal and transversal sections through cardiac muscle cells of WT, *bag3*^*-/-*^ and *bag3*^*-/-*^ + MO-*bag2* embryos at 72 hpf.
(PDF)

S1 Movie Touch evoked assay on *bag3*^*+/+*^ and *bag3*^*+/-*^ embryos showing an adequate response to the touch of the needle. (AVI)

S2 Movie *bag3*^*+/+*^ and *bag3*^*+/-*^ embryos injected with MO-*bag3* do not show an adequate response to the touch of the needle. (AVI)

S3 Movie *bag3*^*-/-*^ embryos injected with MO-*bag2* do not show any flight response to the touch of the needle. (AVI)

S4 Movie *bag3*^*-/-*^ embryos injected with MO-*bag3* show a quick flight response to the touch of the needle. (AVI)

S5 Movie *bag3*^*+/+*^ and *bag3*^*+/-*^ embryos injected with MO-*bag2* show an adequate response to the touch of the needle. (AVI)

S6 Movie *bag3*^*+/+*^ and *bag3*^*+/-*^ embryos injected with MO-*upf*1 show an adequate response to the touch of the needle. (AVI)

S7 Movie *bag3*^*-/-*^ embryos injected with MO-*upf*1 do not show any adequate flight response to the touch of the needle. (AVI)

S8 Movie *bag3*^*-/-*^ embryos show regular cardiac contractility. (AVI)

S9 Movie WT embryos injected with MO-*bag3* show reduced cardiac contractility. (AVI)

S10 Movie *bag3*^*-/-*^ embryos injected with MO-*bag3* show regular cardiac contractility. (AVI)

S11 Movie *bag3*^*-/-*^ embryos injected with MO-*bag2* show reduced cardiac contractility. (AVI)

S12 Movie *bag3*^*-/-*^ embryos injected with MO-*upf*1 show reduced cardiac contractility. (AVI)

S13 Movie WT embryos injected with MO-*bag1* show regular cardiac contractility. (AVI)

S14 Movie *bag3*^*-/-*^ embryos injected with MO-*bag1* show regular cardiac contractility. (AVI)

S1 Table List of the CRISPR RNAsequence, Morpholino sequences and qPCR primers used to perform the experiments described in this work. (DOCX)

S2 Table Excel Worksheet containing the results and analysis of the Proteomic analysis performed on WT and *bag3*^*-/-*^ zebrafish. (XLSX)
