# Supplementary material for: Genetic compensation prevents myopathy and heart failure in an in vivo model of Bag3 deficiency
Source: PLoS Genet. 2020 Nov 2;16(11):e1009088. doi: 10.1371/journal.pgen.1009088 (PMC7605898; doi:10.1371/journal.pgen.1009088)
Supplement: S1 Fig — Overall amino acid identity between human and zebrafish BAG3. (PDF) [file pgen.1009088.s001.pdf]

Suppl. Figure 1

```
BAG3_HUMAN       1 MSA----------ATHSPMMQVASGNGDRDPLPPGWEIKIDPQTGWPFFVDHNSRTTTWN
Bag3_DANRE       1 MAQYSGAKQYQSMKTLSPVETM----ATNDPLPPGWEIKIDPQTGWPFFVDHNNRTTTWN

BAG3_HUMAN      51 DPRVPS-----EGPKETPSSANGPSREGSRLPPAREGHPVYPQLRPGYIPIPVLHEGAEN
Bag3_DANRE      57 DPRHDTKKIFSNGPSMSPETPQDMHK-------TFINEMRQPMLRQGYIPIPVCHENPEP

BAG3_HUMAN     106 R-QVHPFHVYPQPGMQ-RFRTEAAAAAPQRSQSPLRGMPETTQPDKQCGQVAAAAAAQPP
Bag3_DANRE     110 RLQQYPSFSYIHPAVQQNLRTDGRTPSPTPAAH-CRPRSPVQTPSEAC-------SSCSP

BAG3_HUMAN     164 ASHGPERSQSPAASDCSSSSSSASLPSSGRSSLGSHQLPRGYISIPVIHEQNVTRPAAQP
Bag3_DANRE     162 TSHGPEGYQPQGTHQQI-----SGLHQQPRSS--NTGLRAGYIPIPVIHEGAGG---VLP

BAG3_HUMAN     224 SFHQAQKTHYPAQQGEYQTHQPVYHKIQGDDWEPRPLRAASPFRSSVQGASSREGSPARS
Bag3_DANRE     212 S--QLSQSSHPTREKIYREQVPIQ---------IQQNRA---------------ASPI--

BAG3_HUMAN     284 STPLHSPSPIRVHTVVDRPQQPMTHRETAPVSQPENKPESKPGPVGPELPPGHIPIQVI-
Bag3_DANRE     244 QVPLRAQSPVMAQIMGERPQMQQHIGHTAIPSKIEHPVEEI-----IRVPTFEVPIQRVS

BAG3_HUMAN     343 --RKEVDSKPVSQKPPPPSEKVEVKVPPAPVPCPPPSPGPSAVPSSPKSVATEERAAPST
Bag3_DANRE     299 EVPQQIHHQPVQQQQQP-TQQPQPKAQPSP----QVSETSNITIQVPPAPEPQETAAPQT

BAG3_HUMAN     401 APAEATPP--KPGE-AEAPPKHPGVLKVEAILEKVQGLEQAVDNFEGKKTDKKYLMIEEY
Bag3_DANRE     354 -PQEVSSPQLQPEETLEQDLSHPGLVKVQQIVERVEKLAQNVKGFDGKKNDKRYLVLEEM

BAG3_HUMAN     458 LTKELLALDSVDPEGRADVRQARRDGVRKVQTILEKLEQKAIDVPGQVQVYELQPSNLEA
Bag3_DANRE     413 LTKELLALDSVDPEGRPDVRQARRDGVRRVQNILDELEM---------------------

BAG3_HUMAN     518 DQPLQAIMEMGAVAADKGKKNAGNAEDPHTETQQPEATAAATSNPSSMTDTPGNPAAP--
F1Q9J0_DANRE   452 ------IGEMQAGNEAKGEQS--------------------------MIDQLNTERVKEF

BAG3_HUMAN         -
Bag3_DANRE     480 S
```
