# Supplementary figures and images for: Genetic compensation prevents myopathy and heart failure in an in vivo model of Bag3 deficiency
Source: PLoS Genet. 2020 Nov 2;16(11):e1009088. doi: 10.1371/journal.pgen.1009088 (PMC7605898; doi:10.1371/journal.pgen.1009088)

# Suppl. Figure 2

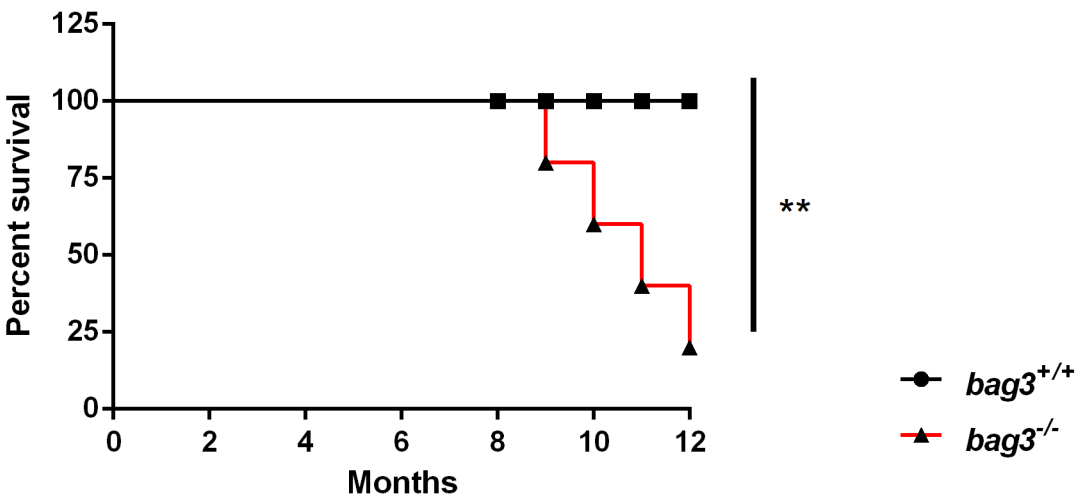

Supplement: S2 Fig — (PDF) [file pgen.1009088.s002.pdf]

# Suppl. Figure 3

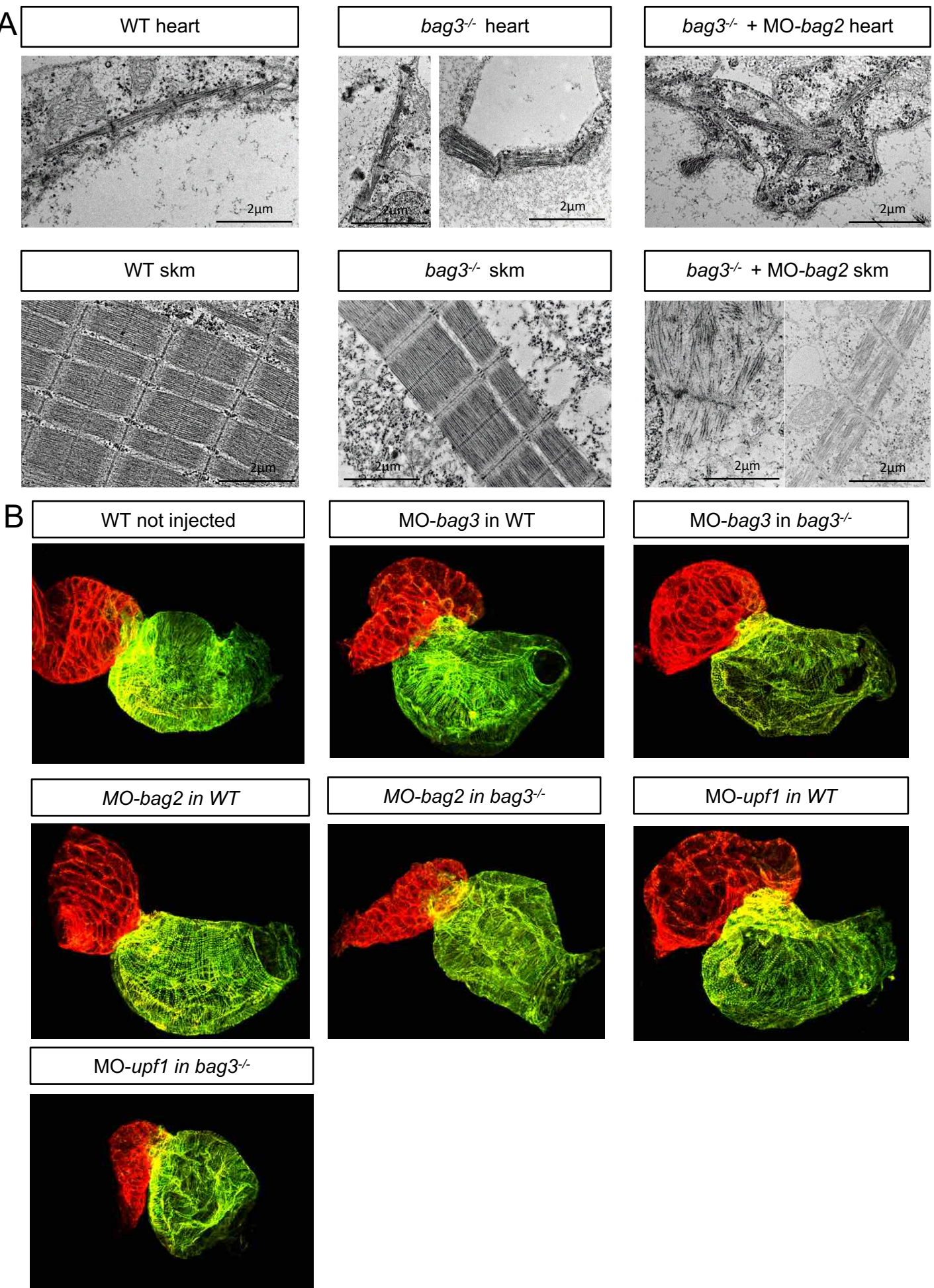

Supplement: S3 Fig — (a) Transmission electron microscopic (TEM) analysis of parasagittal and transversal sections through cardiac and skeletal muscle cells of WT, bag3-/- and bag3-/- + MO-bag2 embryos at 72 hpf. In contrast to the WT and bag3-/-, bag3-/- + MO-bag2 cardiomyocytes and fast-twitch skeletal muscle fibers show disrupted sarcomeric structures. (b) Hearts at 72 hpf stained with chamber-specific myosin antibodies, MF20 (red) and S46 (green) (MF20 marks the entire heart and S46 is atrium specific). Heart chamber specification appears to be normal in all analyzed embyros. (PDF) [file pgen.1009088.s003.pdf]

# Suppl. Figure 4

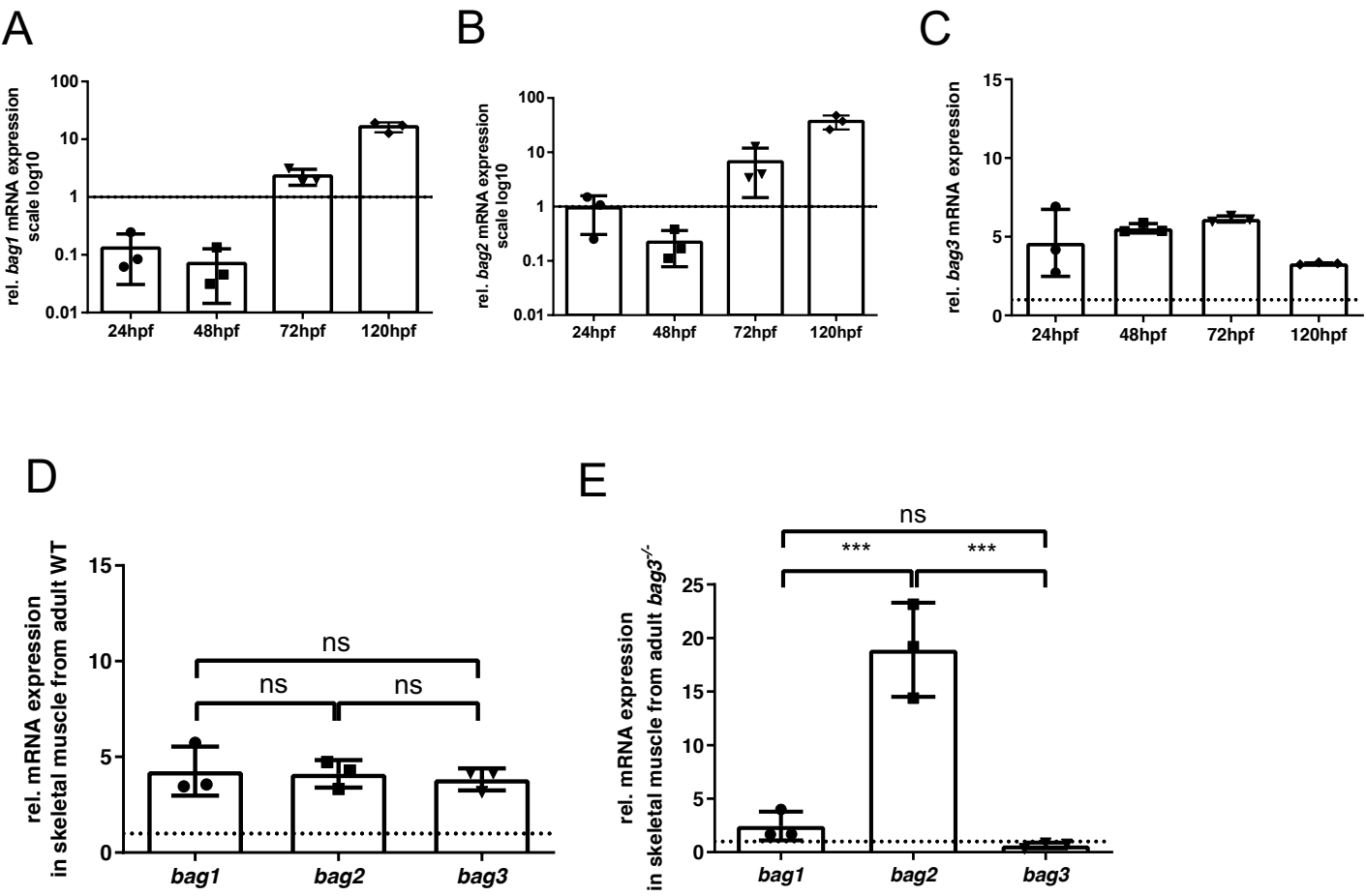

Supplement: S4 Fig — The graph shows a very similar expression pattern for bag1 and bag2. (d) qPCR showing bag1, bag2 and bag3 levels in adult zebrafish skeletal muscles (N = 3, mean±S.D, One-way ANOVA followed by tukey's multiple comparison analysis P = 0.8457). (e) bag2 transcript levels are significantly increased in skeletal muscle of adult bag3-/- zebrafish (N = 3, mean±S.D, One-way ANOVA followed by tukey's multiple comparison analysis ***P = 0.0003). (PDF) [file pgen.1009088.s004.pdf]

# Suppl. Figure 5

A

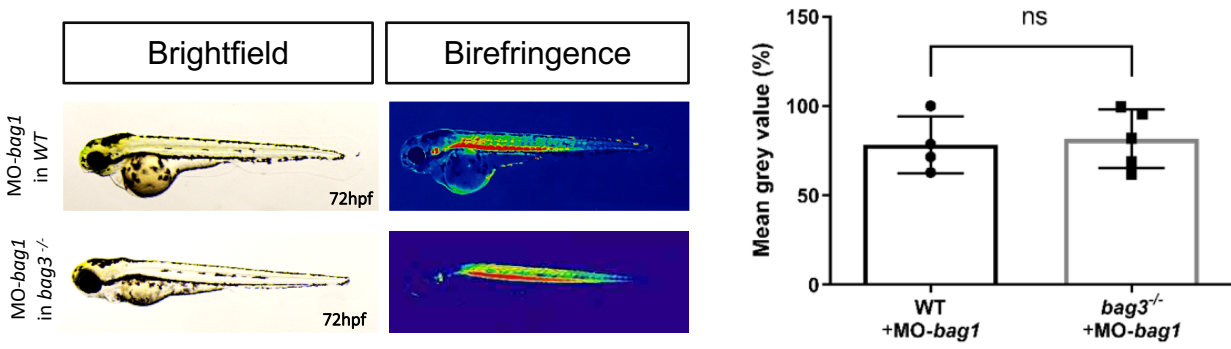

B

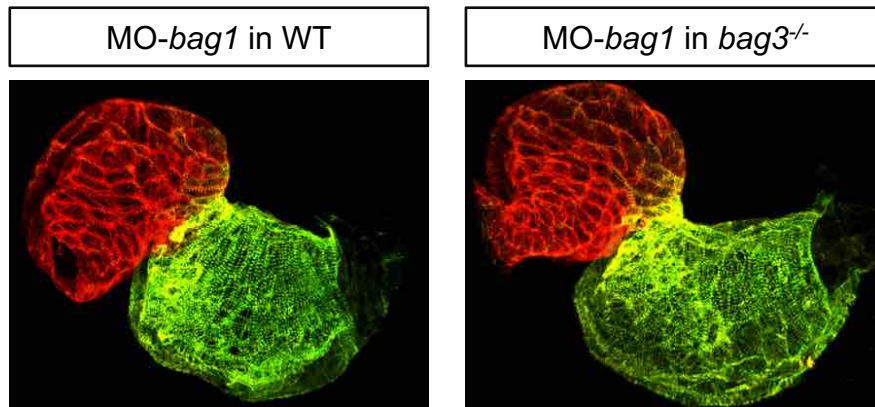

C

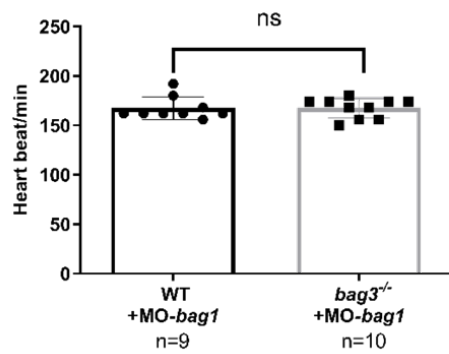

D

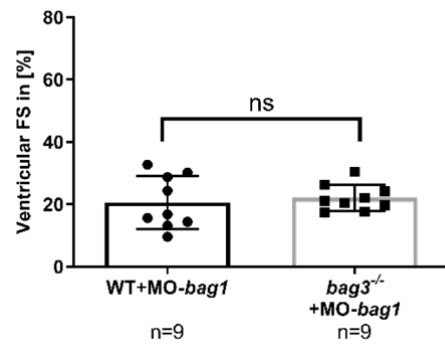

Supplement: S5 Fig — (a) Brightfield and birefringence images of bag1 splice MO injected WT and bag3-/- embryos at 72 hpf don´t show any (cardio)-myopathy phenotype. The densitometric analysis of birefringence signals supports the absence of sarcomeric disorganization. Individual samples are shown (n = 4, P>0.9999 determined using two tailed t-test). (b) MF20 and S46 immunostainings of WT and bag3-/- embryos at 72 hpf injected with MO-bag1 reveal regular specification of the cardiac chambers. (c) Heart rate quantification of bag1 morphants and bag3-/- + MO-bag1 at 72 hpf reveals no functional cardiac impairments (N = 3, n = 9/10. HR MO-bag1 injected WT embryos: 167±11.40 heart beat/min; HR bag3-/- +MO-bag1: 167±9.98 heart beat/min; mean ± S.D. P = 0.9894 determined using two-tailed t-tests). (d) Ventricular FS of bag1 morphants (20.59±8.47%) and bag3-/- + MO-bag1 embryos (FS: 22.12±4.20%) at 72 hpf is unaltered (N = 3, n = 9; Mean± SD P = 0.6373 determined using two-tailed t-tests). (PDF) [file pgen.1009088.s005.pdf]

# Suppl. Figure 6

A

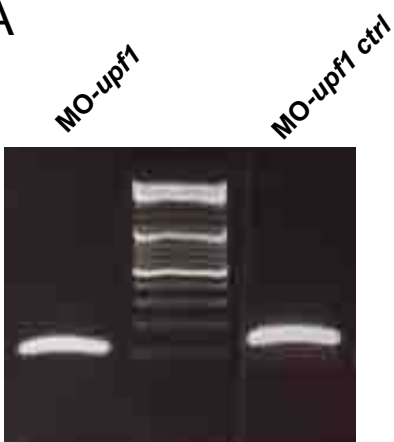

B

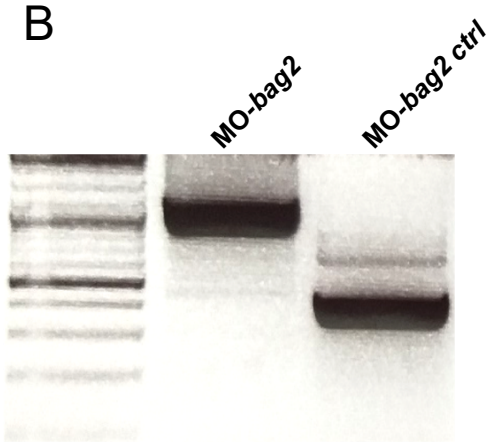

C

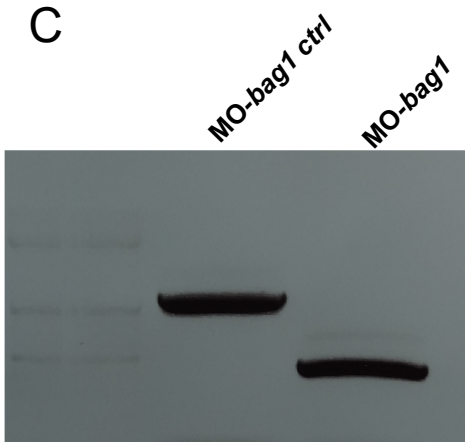

Supplement: S6 Fig — (a) Injection of MO-upf1 (splice-blocking morpholino) results in the partial skipping of upf1 exon 1, leading to a frame shift, a premature stop codon and the premature termination of Upf1 translation [43, 44]. (b) Injection of MO-bag2 (splice-blocking morpholino) results in the integration of the intron 2, a frame shift, a premature stop codon and the premature termination of Bag2 translation. (c) Injection of splice MO-bag1 (splice-blocking morpholino) results in the skipping of the exon 2, a frame shift, a premature stop codon and the premature termination of Bag1 translation. (PDF) [file pgen.1009088.s006.pdf]

# Suppl. Figure 7

WT heart

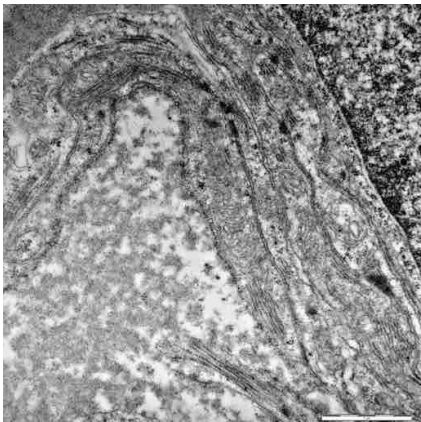

*bag3*<sup>-/-</sup> heart

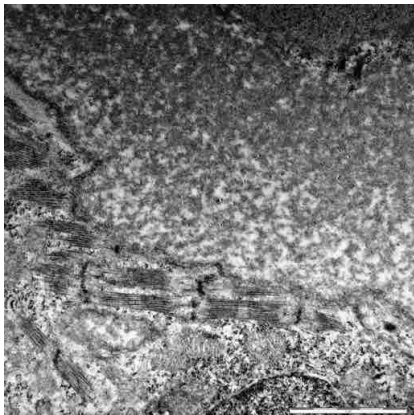

*bag3*<sup>-/-</sup> + MO-*bag2* heart

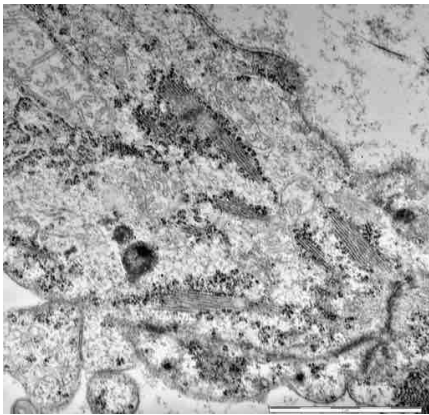

Supplement: S7 Fig — Additional transmission electron microscopic (TEM) pictures of parasagittal and transversal sections through cardiac muscle cells of WT, bag3-/- and bag3-/- + MO-bag2 embryos at 72 hpf. (PDF) [file pgen.1009088.s007.pdf]
