# Supplementary material for: Genetic compensation prevents myopathy and heart failure in an in vivo model of Bag3 deficiency
Source: PLoS Genet. 2020 Nov 2;16(11):e1009088. doi: 10.1371/journal.pgen.1009088 (PMC7605898; doi:10.1371/journal.pgen.1009088)
Supplement: S1 Table — (DOCX) [file pgen.1009088.s022.docx]

|  | Sequence (5‘-3‘) |
| --- | --- |
| *bag3* Exon2 crRNA | GTCATGAAAACCCTGAACCCAGG |
| uni-tracrRNA | AAACAGCAUAGCAAGUUAAAAUAAGGCUAG  UCCGUUAUCAACUUGAAAAAGUGGCACCGAGUCGGUGCU |
| *bag3*_fwd | CAGGACATGCACAAAACCTTTA |
| *bag3*_rev | CAGGACATGCACAAAACCTTTA |
| MO-*bag3* e2i2 splice | GCTTTCTCATGATCTTACCTCAGGC |
| MO-*bag3*_5 mispair | GCTAACTCATGCTCTTACAACAGGC |
| MO-*bag2* e2i2 splice | AGGCAGAACTGAAGTGTTACCGTCA |
| MO-*bag2*_5 mispair | AGCCACAAGTGAAGTCTTACCCTCA |
| MO-*upf1* splice blocking | TTTTGGGAGTTTATACCTGGTTGTC |
| MO-*upf1*_5 mispair | TTTTCGGACTTTATAGCTGCTTCTC |
| MO-*bag1* i1e2 splice | TGTGCCTGAGACAAGAACATTCAAT |
| MO-*bag1*_5 mispair | TGAGCCTGAGACACCAACATCAAAT |
| *bag3_*qRT_fwd | GGCTCAGATCATGGGAGAGA |
| *bag3_*qRT_rev | TGAGGCTGCTGTGTAGGTTG |
| *bag2_*qRT_fwd | GAAGTTTCAGAGCGTGGTGA |
| *bag2_*qRT_rev | CCAGGCGTCTCTTGATCTTC |
| *bag1_*qRT_fwd | GGGGTACTTAGCGCAGTGTG |
| *bag1_*qRT_rev | CTGAATCTTGGCCAGGTGAT |
| *β-actin*_qRT_fwd | GCAGAAGGAGATCACATCCCTGGC |
| *β-actin*_qRT_rev | CATTGCCGTCACCTTCACCGTTC |
| *rpl13_*qRT_fwd | TCTGGAGGACTGTAAGAGGTATGC |
| *rpl13_*qRT_rev | AGACGCACAATCTTGAGAGCAG |
